# Sex-specific transcriptional and proteomic signatures in schizophrenia

**DOI:** 10.1038/s41467-019-11797-3

**Published:** 2019-09-02

**Authors:** Jari Tiihonen, Marja Koskuvi, Markus Storvik, Ida Hyötyläinen, Yanyan Gao, Katja A. Puttonen, Raisa Giniatullina, Ekaterina Poguzhelskaya, Ilkka Ojansuu, Olli Vaurio, Tyrone D. Cannon, Jouko Lönnqvist, Sebastian Therman, Jaana Suvisaari, Jaakko Kaprio, Lesley Cheng, Andrew F. Hill, Markku Lähteenvuo, Jussi Tohka, Rashid Giniatullin, Šárka Lehtonen, Jari Koistinaho

**Affiliations:** 1 Department of Clinical Neuroscience, Karolinska Institutet, Byggnad R5, SE-171 76 Stockholm, Sweden; 2 Department of Forensic Psychiatry, University of Eastern Finland, Niuvanniemi Hospital, Niuvankuja 65, FI-70240 Kuopio, Finland; 3 A.I. Virtanen Institute for Molecular Sciences, University of Eastern Finland, PO Box 1627, FI-70211 Kuopio, Finland; 4 Neuroscience Center, University of Helsinki, PO Box 63, FI-00271 Helsinki, Finland; 5 Department of Pharmacology, University of Eastern Finland, PO Box 1627, FI-70211 Kuopio, Finland; 6 Department of Psychology and Psychiatry, Yale University, 1 Prospect Street, New Haven, Connecticut 06511 USA; 7 Mental Health Unit, Department of Public Health Solutions, National Institute for Health and Welfare, PO Box 30, FI-00271 Helsinki, Finland; 8 Department of Psychiatry, University of Helsinki, PO Box 22, FI-00014 Helsinki, Finland; 9 Department of Mental Health and Substance Abuse Services, National Institute for Health and Welfare, PO Box 30, FI-00271 Helsinki, Finland; 10 Department of Public Health, University of Helsinki, PO Box 20, FI-00014 Helsinki, Finland; 11 Institute for Molecular Medicine FIMM, University of Helsinki, PO Box 20, FI-00014 Helsinki, Finland; 12 Department of Biochemistry and Genetics, La Trobe Institute for Molecular Science, La Trobe University, Science Drive, Bundoora, VIC 3083 Australia

**Keywords:** Stem cells, Diseases

## Abstract

It has remained unclear why schizophrenia typically manifests after adolescence and which neurobiological mechanisms are underlying the cascade leading to the actual onset of the illness. Here we show that the use of induced pluripotent stem cell-derived neurons of monozygotic twins from pairs discordant for schizophrenia enhances disease-specific signal by minimizing genetic heterogeneity. In proteomic and pathway analyses, clinical illness is associated especially with altered glycosaminoglycan, GABAergic synapse, sialylation, and purine metabolism pathways. Although only 12% of all 19,462 genes are expressed differentially between healthy males and females, up to 61% of the illness-related genes are sex specific. These results on sex-specific genes are replicated in another dataset. This implies that the pathophysiology differs between males and females, and may explain why symptoms appear after adolescence when the expression of many sex-specific genes change, and suggests the need for sex-specific treatments.

## Abstract (web summary)

Noise due to genetic heterogeneity potentially impacts the the discovery of genes that contribute to diseases such as schizophrenia (SCZ). In this study, authors minimize the disease-irrelevant noise between SCZ and healthy individuals by profiling transcriptional signatures among discordant monozygotic twin pairs, and demonstrate that although sexes share many of the final common pathways, the underlying primary pathophysiology of SCZ differs between males and females.

## Introduction

It has been estimated that about 50% of the risk of schizophrenia is attributable to DNA sequence variation and the rest is explained by epigenetic mechanisms modified by the environment^1^. Studies on monozygotic twin pairs have shown that if one twin has schizophrenia, the risk of illness for the co-twin is about 50% and the risk of disease decreases with decreasing genetic similarity for relative pairs. Hundreds of genes contribute to the risk of schizophrenia and it has been difficult to find molecular mechanisms to explain the illness phenotypes^2^. Large studies comparing various mental disorders have shown disease specificity of gene expression profiles in post-mortem cerebral cortex between schizophrenia and bipolar disorder^3,4^. Whether these abnormalities are related purely to illness process or are also due to effects of treatment exposures and other secondary factors found at the endpoint is difficult to resolve, but data from animal models implied that most psychotropic medications had little effect on the transcriptome^4^.

Pathophysiology of psychiatric diseases can be modeled using induced pluripotent stem cell (iPSC)-derived neurons. Studies on iPSC-derived neurons carried out in small numbers of individuals suggest that cAMP and WNT signaling pathways, neuronal differentiation, and synaptic functions^5^ may be altered in familial schizophrenia and in patients harboring penetrant genetic variants. It has been suggested that, because of noise due to genetic heterogeneity, the number of iPSC study individuals with schizophrenia should be increased substantially to detect truly significant findings^6^. Here we minimize the disease-irrelevant noise between affected and healthy individuals in iPSC-derived neurons by studying disease-discordant monozygotic twin pairs. We identify factors that are associated with the shared risk of schizophrenia among monozygotic twins, and molecular pathways and neuronal electrophysiological abnormalities that are related to the actual onset of the illness.

## Results

### Transcriptional signatures related to familial risk

**Fig. 1:**
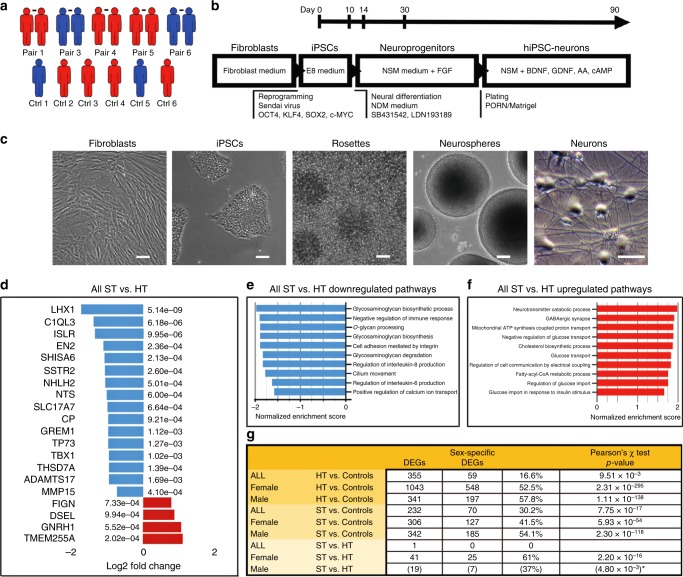
RNA expression analysis of affected (ST) and unaffected (HT) twins using hiPSC-derived neurons. **a** Sex breakdown of monozygotic twin pairs and control individuals of the study (females in red; males in blue). **b** A flow chart of the reprogramming and neural differentiation process. **c** Bright-field images of fibroblasts, iPSCs, rosettes, expanded neurospheres, and mature neurons. Scale bar 50 µm. **d** Top list of differently expressed genes (DEGs) for all affected (ST) vs. unaffected (HT) twins and enriched (**e**) down- and (**f**) upregulated pathways. **g** Summary of DEGs and proportion of sex-specific DEGs. DEGs cutoffs: adjusted *p*-value < 0.05 and at least twofold (i.e., onefold log2 change) up- or downregulation. *Fisher’s exact test

**Fig. 2:**
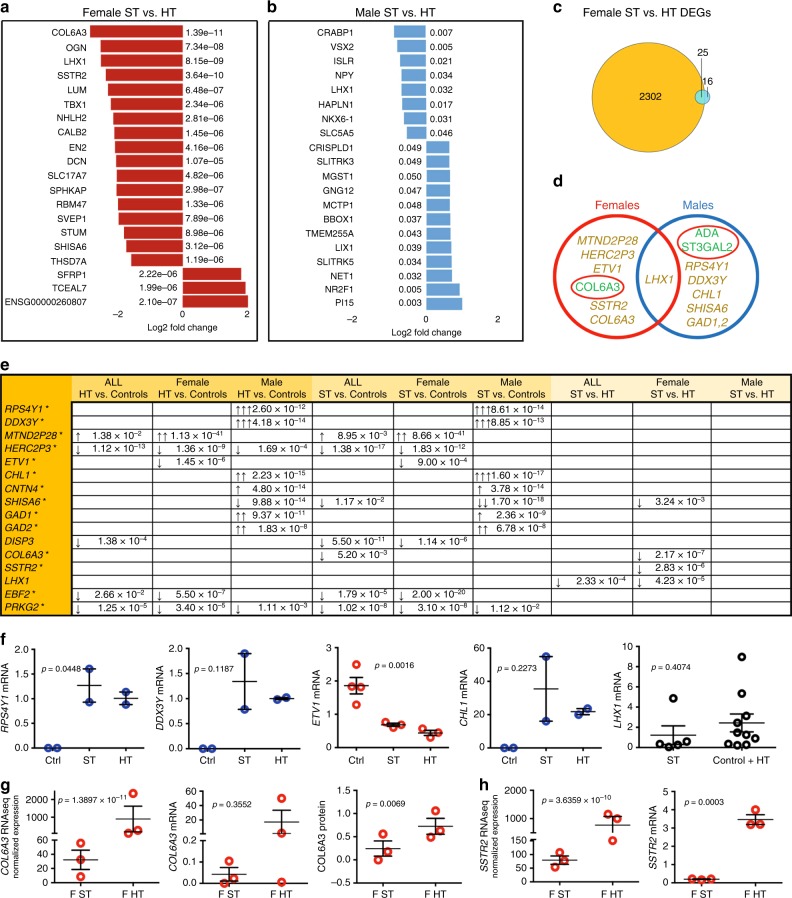
RNA expression analysis of twin pairs using hiPSC-derived neurons. Top list of genes for the following comparison: (**a**) female and (**b**) male affected (ST) and unaffected (HT) twins. **c** The proportion of sex-specific DEGs (overlay of yellow and blue). **d** The overlap of the findings among males and females. Illness-specific genes are shown in yellow and proteins in green, respectively. **e** Adjusted *p*-values for presented genes through different comparison sets. **f** qPCR validation of a top list of genes from several comparison sets, separately presented for (**g**) *COL6A3* gene and protein (data presented from RNAseq, qPCR, and proteomics) and (**h**) *SSTR2* gene. The expression of genes in female (red circles), in males (blue circles), and in both (black circles). The error bars indicate SEM. The log2-fold change indicated as follows: ↑↑↑ for > 10-fold, ↑↑ for 5–10-fold, and ↑ for 1–5-fold increase; *sex-specific genes

We generated and fully characterized iPSC lines from six healthy controls with the lowest possible Positive and Negative Syndrome Scale (PANSS) score 30 and five discordant monozygotic twin pairs (two pairs with family history of schizophrenia) with PANSS scores ranging from 30 to 49 in healthy (unaffected, indicated as HT) twins and from 53 to 113 in affected (indicated as ST) twins (Supplementary Table 1 and Supplementary Figs. 1–4). Three of the ST twins were females who had a response to clozapine, an atypical antipsychotic drug used for treatment-resistant schizophrenia, and the two male patients were treated with first-line antipsychotics (Supplementary Table 1). We chose to differentiate the cells into cortical neurons expressing markers of GABAergic and glutamatergic neurons, because they are among the most affected cells in schizophrenia (Fig. 1). Figure 1g shows the number of differentially expressed genes (DEGs) in the comparison between unaffected twins and healthy controls (associated with familial risk of schizophrenia without clinical illness), between ST twins and healthy controls (associated with both familial risk and clinical illness), between ST and unaffected twins (associated purely with clinical illness), and between male and female controls. The genes with the most robust differences are shown in Fig. 2e and the whole set of DEGs are shown in Supplementary Data 1–10. *RPS4Y1* and *DDX3Y* showed the strongest signal for shared familial risk among male twins and the effect sizes for the upregulated expression of these Y chromosome genes were extremely large. In addition, *CHL1*, *CNTN4*, *Shisa6*, *GAD1*, and *GAD2* showed a strong signal for familial risk among males. In the comparison between healthy control males and females, *ETV1* was the first and *CHL1* was the third among the total of 19,462 genes in the rank order list showing the most significant differences between sexes (indicating sex specificity, Supplementary Data 10).

### Transcriptional signatures related to clinical illness

To study how gene expression is associated with actual clinical illness, we compared ST twins with their healthy co-twins and found decreased expression of *LHX1*, a transcription factor previously linked to schizophrenia^7,8^ (Fig. 1d). Also over 800 pathways were enriched in ST twins when compared with their co-twins. Especially, pathways related to glycosaminoglycan metabolism were downregulated, whereas pathways involving neurotransmitter catabolism and GABAergic synapse were upregulated (Fig. 1e, f and Supplementary Data 11). In the comparison between ST vs. unaffected twins, the differently expressed genes (DEGs) and enriched pathways were different between males and females (Fig. 2a, b, 1e, f, and Supplementary Data 8, 9, 12, 13). Between ST and unaffected female twins, the genes with highly significant expression changes included *COL6A3*, as well as *SSTR2* and *LHX1*. (Fig. 2a, b, f–h). In men, with only two twin pairs, none of the gene expressions survived correction for multiple comparisons; however, several genes, including *LHX1*, showed nominally significant difference in the comparison between ST vs. unaffected twins (Fig. 2b). When ST twins were compared with healthy twins, the enriched pathways in females included, among others, pathways of neural cell development, neural differentiation, and glycans (Supplementary Data 12), whereas in males it included a large proportion of the enriched pathways related, among others, to Wnt signaling, mitochondrion respiration, and metabolic processes (Supplementary Data 13).

### Sex-specific gene expression

As sex appeared to be a major determinant of gene expression changes related to schizophrenia, we also compared the females with the males among the healthy controls and identified 2327 genes (Benjamini–Hochberg corrected *p*-value < 0.05) with up to 246-fold (7.94 log2-fold) difference in gene expression, and 964 significantly enriched pathways (Supplementary Data 10 and 14). Thus, 2327 genes from all 19,462 (12%) detectable genes showed sex-specific expression. The Venn diagram (Fig. 2c) shows that within the 41 genes that significantly differ by expression in female ST twins from female unaffected twins (blue), 25 genes (61.0%, *p* = 2.2 × 10^−16^, Pearson’s *χ*^2^- test, for the difference between proportions, Fig. 1g) belong to sex-specific genes. In the comparison of ST vs. unaffected male twins, no genes with significantly different expression were identified when correction for multiple comparisons was applied. However, 7 of the 19 (37%) genes with nominal significance were sex-specific (*p* = 4.8 × 10^−3^, Fisher’s exact test). These data indicate that a large proportion of illness-related genes are sex-specific (Fig. 1g). Although males and females share many of the final molecular pathways in schizophrenia, the underlying primary pathophysiology of schizophrenia obviously differs between males and females, and may contribute to sex-dependent features of the disease. As all three female patients had treatment-resistant schizophrenia (TRS) and were treated with clozapine, it is apparent that also the type of severity of the illness may contribute to the observed alterations in gene expression. Figure 1g and the Venn diagram in Fig. 2d summarize the illness-related DEGs and proteins among males and females, showing *p*-values down to 2.3 × 10^−295^.

### Reproducibility

We replicated our results by using the dataset by Hoffman et al.^6^. Supplementary Table 8 shows the comparisons of number of DEGs between datasets. Supplementary Data 15–18 show the DEGs in hiPSC-derived neurons and Supplementary Data 19–22 show the DEGs in hiPSC-derived neural progenitor cells (NPCs) in the Hoffman et al.^6^ dataset. Both datasets show that the number of DEGs is larger in the comparison between male patients vs. male controls and between female patients vs. female controls than when all patients are compared with all controls, despite lower number of subjects including males only or females only. This may be explained by the fact that DEGs in male and female comparisons are not the same and the results are diluted or counterbalanced when males and females are analyzed together. Therefore, the number of DEGs gets smaller despite the higher number of subjects.

Venn diagram in Supplementary Fig. 5 shows the overlap of sex-specific genes (healthy males vs. females) in neurons in our dataset and in neurons and NPCs in the Hoffman et al.^6^ dataset. The number of sex-specific genes was smaller (244) in hiPSC-derived neurons in the Hoffman et al.^6^ dataset (with NPC-derived neurons differentiated for 6 weeks) than in our hiPSC-derived neurons (NPC-derived neurons differentiated for 10 weeks), with 18 genes overlapping with our sex-specific genes. The corresponding numbers were 114 and 6 concerning hiPSC-derived NPCs. This suggests that the number of sex-specific genes increases as a function of time during maturation of cells.

Supplementary Table 9 shows the proportion of sex-specific genes in neurons and NPCs in Hoffman et al.^6^ dataset. Also in that dataset, proportion of sex-specific genes was much higher than expected concerning DEGs in the comparison between all patients with schizophrenia vs. all controls (5.0% vs. 0.6%, *p* = 4.6 × 10^−4^), between female patients vs. female controls (10.2% vs. 0.6%, *p* = 1.5 × 10^−38^, and between male patients vs. male controls (21.8% vs. 0.6%, *p* = 6.7 × 10^−112^). The results from NPCs were well in line with the results from neurons (*p*-values 4.1 × 10^−6^, 9.8 × 10^−74^, and 2.7 × 10^−100^, respectively). The results on schizophrenia:sex interaction for sex-specific DEGs are shown in Supplementary Table 10. The statistically significant DEGs are shown in Supplementary Data 23–26.

### Proteomic analyses

**Fig. 3:**
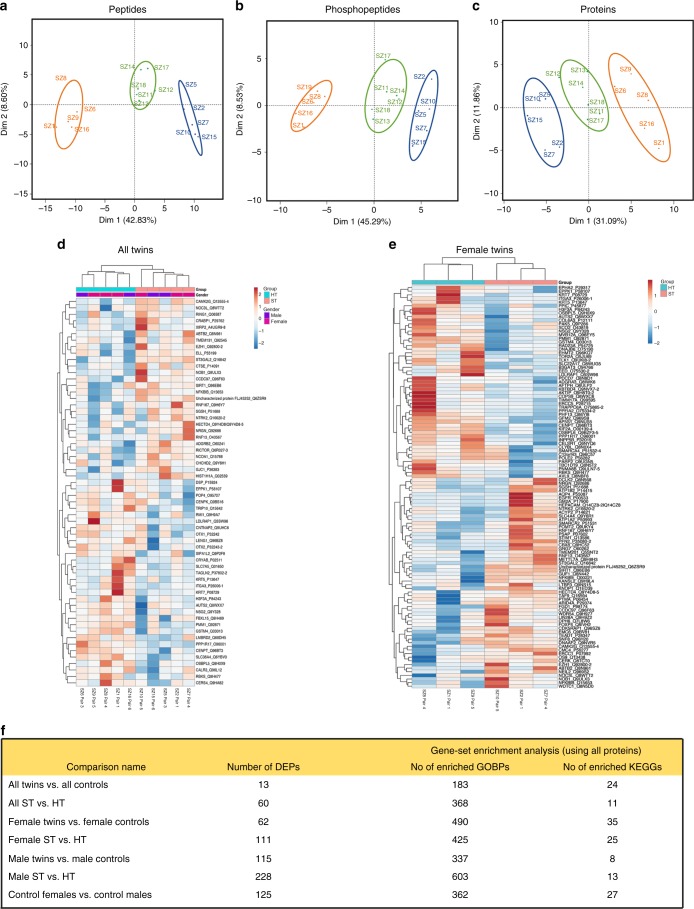
Proteomic analysis of unaffected (HT) and affected (ST) twins using hiPSC-derived neurons. PCA analysis of (**a**) peptides, (**b**) phosphopeptides, and (**c**) proteins data after feature selection. PC1 (Dim 1) vs. PC2 (Dim 2) shows the separation of the data into affected twins (blue circle) and unaffected twins (orange circle) mainly on PC1. The control group is also included (green circle). PCA plots were generated using subsets of differentially expressed features (peptides and phosphopeptides: *p* < 0.01; proteins: *p* < 0.05). **d** Heat maps of differentially expressed proteins when ST compared with HT twins (five pairs) and **e** between female twins. **f** Summary of differently expressed proteins (DEPs) and enriched pathway analyses with cutoffs: unadjusted *p*-value <  0.05. PCA plots were generated after feature selection, using differentially expressed features from the comparison of affected (ST) twins and unaffected (HT) twins with statistical significance levels of *p* < 0.01 for peptides and phosphopeptides and *p* < 0.05 for protein data

Messenger RNA and corresponding protein levels in cells correlate poorly due to variation in processes controlling steady-state mRNA or protein abundances. To investigate whether protein expression is changed in cortical neurons of monozygotic twins discordant for schizophrenia, proteomic analysis was performed at the peptide, phosphopeptide, and protein levels in pairwise comparisons (Fig. 3a–c).

In the comparison of all 5 ST and 5 unaffected twins, we found 60 proteins such as CAMK2G, PPP1R17, phosphomannomutase 1, ST3GAL2, and SLC36A4 (Fig. 3d and Supplementary Data 27) with differential expression and 379 enriched pathways with nominal significance (Fig. 3f and Supplementary Data 28). Similar to the transcriptomic study, running proteomic analyses separately in females and males revealed more differentially expressed schizophrenia-associated proteins and enriched pathways than when sexes were pooled together (Supplementary Data 29, 30). In females, ST twins differed from healthy co-twins by 111 proteins (Fig. 3e, f) and 450 pathways with nominal significance. The most consistent finding among females was upregulation of CAMK2G, differentiating ST twins from unaffected co-twins (Supplementary Data 29). Correspondingly, in males, ST twins differed from their unaffected co-twins by 228 differentially expressed proteins and 616 enriched pathways (Fig. 3d–f). Of these proteins, adenosine deaminase (ADA) and ST3GAL2 showed the most robust change, surviving correction for multiple comparisons (Supplementary Data 30).

Differentially expressed proteins did not correspond to DEGs in cortical neurons derived from monozygotic twins from pairs discordant for schizophrenia, with the exception of downregulation of *COL6A3* and COL6A3 (Fig. 2g), which was the most robust finding in gene expression in comparison between ST vs. unaffected female twins, and which showed a nominal *p*-value of 6.9 × 10^−3^ in corresponding proteomic analyses. Only COL6A3 gene–protein pair remained significant with Bonferroni-corrected threshold *p* < 0.05 when studying only the genes for which the corresponding protein differences were significant at *p* < 0.01 (Supplementary Data 31). In conclusion, drastic changes in schizophrenia in both protein and gene expression were discovered and linked to central nervous system development and various other pathways in a sex-specific manner. Moreover, both mRNA and protein expression in iPSC-derived cortical neurons were distinct in healthy males and females (Supplementary Data files 10 and 32). Observed gene and protein expression differences were not explained by copy number variants (see Supplementary Tables 3–6 for details).

### Electrophysiological analyses

**Fig. 4:**
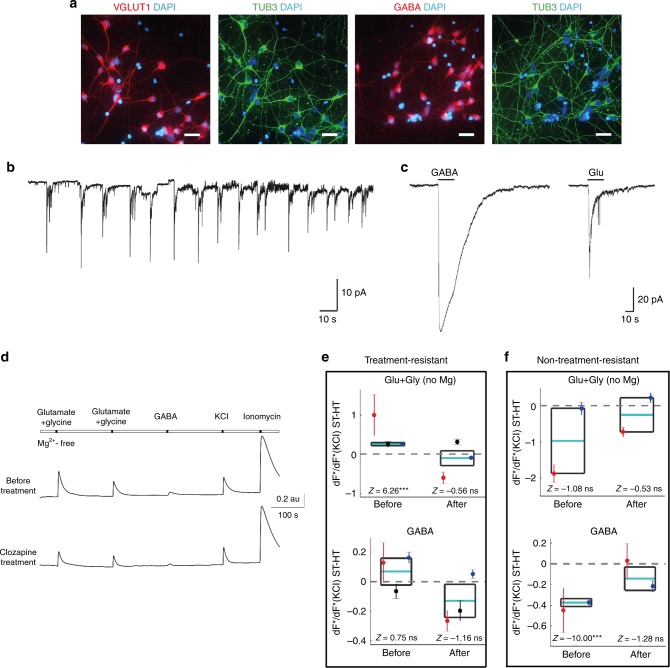
Spontaneous neuronal activity and neuronal calcium imaging of affected and unaffected twins. **a** hiPSC-derived neurons stained for VGLUT1 (red), TUB3 (green), and GABA (red). **b** Regular spontaneous neuronal reactivity and (**c**) membrane current induced by 100 µM GABA or by 100 µM glutamate with 10 µM co-agonist glycine in neuronal cultures of healthy control subjects. Scale bar 20 µm. **d** Representative calcium traces and quantification from neurons of (**e**) treatment-resistant and (**f**) non-treatment-resistant (patients that respond to standard antipsychotics) schizophrenia before and after clozapine treatment. Glu + Gly (no Mg) indicates NMDA-specific responses. ST, affected twin; HT, unaffected twin. In **e** and **f**, results are presented representing both levels of the hierarchical two-level random effects analysis. The cyan line and black box represent, respectively, the mean and its SE of the upper, population level to which *p*-values and *Z*-statistics refer to. Colored dots and their whiskers refer to the first-level analyses of individual subject pairs. Dots represent the average differences between the response between healthy and affected twin. Whiskers represent the SEs of the averages. Population level mean (cyan line) is the inverse variance weighted mean of the subject-pair-wise average differences. The subject pairs are in **e** Red = Pair 1, Black = Pair 4, Blue = Pair 5. **f** Red = Pair 3, Blue = Pair 6. ***p < 0.001

Clozapine is used to treat patients with schizophrenia that do not respond to standard antipsychotic treatment and are classified as having treatment-resistant schizophrenia (TRS). The mechanism of action of clozapine is not exactly known, but the drug regulates several neurotransmitter systems and interacts with GABA and NMDA receptor-mediated glutamatergic signaling. Both NMDA receptors and GABAergic interneurons have been associated with the pathology of schizophrenia and dysfunction of NMDA receptor is considered a major mechanism explaining the symptoms of schizophrenia^9^. As our transcriptomic and proteomic data revealed alterations in genes and proteins regulating GABAergic neurons and glutamatergic pathways, we next compared calcium responses with GABA and glutamate in neurons between the twins with TRS and the twins responding to standard antipsychotics and their healthy co-twins (Supplementary Table 2). As iPSC-derived neurons at this phase correspond to the developmental stage of the early second trimester of pregnancy, GABA_A_ response has a depolarizing effect in our cultures. Glycine without magnesium was added together with glutamate, to preferentially stimulate NMDA receptors. In twin pairs of TRS, there was no significant difference in calcium response to GABA between healthy and ST twins either before or after clozapine treatment (Fig. 4e), whereas, in twin pairs with non-TRS cases, the response was significantly smaller in ST twins before but not after clozapine treatment (Fig. 4f). However, NMDA receptor-mediated calcium response to glutamate in twin pairs of TRS was significantly greater in ST twins before but not after clozapine treatment, whereas in twin pairs of non-TRS the response was similar both before and after clozapine treatment (Fig. 4d–f). These data suggest that regulation of neuronal calcium responses is differentially disturbed in TRS and in schizophrenia responding to standard antipsychotics, and that clozapine treatment may abolish the altered neuronal calcium response to GABA and glutamate in embryonic state neurons of individuals with schizophrenia. As all patients with TRS were females, it is possible that the findings are at least partially attributable to sex-specific differences and this issue should be studied further among male patients with TRS.

## Discussion

The method of using iPSC-derived neurons from pairs of monozygotic illness-discordant twins minimized genetic background heterogeneity and the disease-irrelevant noise in transcriptomic and proteomic analyses. Therefore, this kind of analysis of individual-specific iPSC-derived neurons may be a strategy for early prevention, testing, and development of novel pharmacological treatments. Most of the genes showing the largest effect sizes were different among males and females and had sex-specific expression among healthy controls. These results were confirmed in another hiPSC dataset. This suggests that although sexes share many of the final common pathways involving the same proteins, the underlying primary pathophysiology of schizophrenia differs between males and females. This may explain why the symptoms typically appear after puberty when the expression levels of many sex-specific genes change. The findings of our study are analogous to results by Labonté et al.^10^ which showed marked sexual dimorphism at the transcriptional level in major depressive disorder and suggested that the treatments should be sex-specific due to different pathophysiology between males and females. Our results are also in line with a recent study which observed that men and women have different types of lifespan calendars of gene expression, explaining the differences in the phenotypes and the ages of onset in schizophrenia^11^.

In the analysis of familial risk, Y chromosome genes *RPS4Y1* and *DDX3Y* revealed very large upregulation in the gene expression. Both of these genes are candidate biomarkers for Parkinson’s disease^12^. Also, *CHL1*, *CNTN4*, *Shisa6*, *GAD1*, and *GAD2* showed large effect sizes. A large number of studies have linked *CHL1* and *CNTN4* with schizophrenia, while *CHL1* has also been associated with loss of parvalbumin-expressing hippocampal GABA interneurons^13^, and *CNTN4* with antipsychotic responses^14^. *Shisa6* has been reported in several studies to prevent desensitization of AMPA-type glutamatergic receptors during synaptic activity^15^. A large number of studies have linked glutamic acid decarboxylase genes *GAD1* and *GAD2* with schizophrenia^16^.

When all ST twins were compared with all HT twins, downregulation of *LHX1* was the only finding surviving correction for multiple comparisons. *LHX1* is highly expressed in the developing brain. It modulates survival and migration of GABAergic interneurons^17^ and regulates sleep timing by light, making the gene a candidate genetic factor contributing to schizophrenia. In the comparison restricted to females, *COL6A3*, *SSTR2*, and *LHX1* showed the most robust findings. *COL6A3* has previously been linked to brain white matter abnormalities^18^ and *SSTR2* (somatostatin receptor 2) has previously been reported to both be associated with schizophrenia^7,9^ and also to be the most robust biomarker in post-mortem studies^19^.

In the pathway analyses, the actual illness was associated with altered *N*-glycan synthesis, CAMK2G, GABAergic synapse, and purine metabolism. Blocking of NMDA receptors results into full-blown schizophrenic symptoms in healthy individuals and NMDA dysfunction is considered as a major pathophysiological factor of the illness^9^. Recently, it has been observed that activation of NMDA-type glutamate receptors leads to input-specific long-term potentiation of dendritic inhibition mediated by somatostatin-expressing interneurons. This form of plasticity is expressed postsynaptically and requires both CaMKIIα and the β2-subunit of the GABA-A receptor^20^. Our results suggest that somatostatin 2 receptor defect may be related to dysfunction of NMDA receptors in somatostatin/calreticulin GABAergic interneurons. It must be acknowledged that GABA is excitatory until the third trimester of gestation and only then becomes an inhibitory transmitter when the KCC2/NKCC1 balance of chloride transporters changes^21^. Our results suggest that among individuals affected with TRS, the NMDA receptor pathway is overactive in embryonic-state neurons during the second trimester, although it may be downregulated and associated with dopaminergic defects later in life. *N*-glycans, calnexin, and calreticulin in the endoplasmic reticulum together are important in protein folding in eukaryotic cells^22^, and CaMK signaling in embryonic stem cells is dependent on calreticulin. Our results showed consistent downregulation of both *N*-glycan and calnexin/calreticulin pathways in shared genetic risk and an actual illness, implying their essential role in schizophrenia. Our findings on downregulated glycosaminoglycan metabolism are in line with a large number of studies, indicating that deficits in perineural nets (PNNs), glycosaminoglycan-rich extracellular matrix structures, are important in the pathophysiology of schizophrenia^23–25^. PNNs regulate structural and functional synaptic plasticity and typically assemble around fast-spiking interneurons implicated in learning and memory^23–25^. PNNs and extracellular matrix form the tetrapartite synapse, which has been suggested to be a key concept in the pathophysiology of schizophrenia^23^.

In proteomic analyses, large effect sizes were observed for CAMK2G, a component of calcium/calmodulin-dependent protein kinase type II that has been reported to be involved in schizophrenia^26^, PPP1R17 (DARPP32)^27^, the most important integrator between cortical input and the basal ganglia, and phosphomannomutase 1^28^, an enzyme necessary for *N*-linked glycosylation and secretion of glycoproteins. It has also been observed that treatment of schizophrenia with olanzapine results in altered glycosylation of serum glycoproteins^29^. Also ST3GAL2, which regulates sphingolipid metabolism, and SLC36A4, a high-affinity transporter for proline and tryptophan, were altered in ST twins (Supplementary Data 14). The functions related to these proteins have been previously reported to be abnormal in schizophrenia^30–33^.

The only proteins surviving correction for multiple comparisons were ADA and ST3GAL2 in the male pairs. ADA is a peripheral biomarker of schizophrenia correlating with the antipsychotic efficacy of clozapine and involved in the purine metabolism pathway^34^. ST3GAL2 is a sialyltransferase gene responsible for sialylation of gangliosides and glycoproteins, and knockout of this gene results into profound cognitive disability^35^, and a recent study suggests that altered sialylation and glucosylation contribute to the increased risk of schizophrenia^36^. Among the top proteins approaching statistical significance when corrected for multiple comparisons was OTX2, a transcription factor regulating development of parvalbumin-immunoreactive GABAergic interneurons that are decreased in schizophrenia^37^.

To our knowledge, this is the first study to investigate calcium responses to GABA and glutamate exposures in iPSC-derived neurons in schizophrenia. We observed abnormal calcium responses to NMDA-specific glutamate or GABA exposure in embryonic neurons of ST twins compared with their healthy twins and the differences disappeared with clozapine treatment. This implies that early clozapine type of treatment might stop the cascade leading to the development of full-blown illness. However, our results were based on a small number of subjects and should be interpreted with caution.

In conclusion, the use of discordant monozygotic twins can minimize noise due to genetic heterogeneity and enhance illness-specific signal. Our results indicate that the neurobiological pathophysiology of schizophrenia differs between males and females, and suggest the need for sex-specific treatments.

## Method

### Study subjects

A total of 6 monozygotic twin pairs discordant for schizophrenia (SZ) that were previously well-characterized by imaging and clinical history (more than 20 publications, see, e.g. ^38,39^), as well as 6 non-related age-matched controls were included in this study. The sociodemographic and clinical characteristics of the patients are shown in Supplementary Table 1. Each subject was diagnosed and assessed by a trained psychiatrist according to the Diagnostic and Statistical Manual of Mental Disorders, fourth edition criteria based on a structured clinical interview. Symptoms of schizophrenia were assessed using the PANSS scale around the time of skin sampling (Supplementary Table 1). The project has been approved by the Ethics Committee of the Helsinki University Hospital District, license number 262/EO/06. Informed consent was obtained from all study subjects. One pair of twins was excluded from the study based on PANSS score of the index twin that did not differ either from unaffected twin or healthy controls.

### Generation of hiPSCs and its characterizations

Skin biopsy-derived fibroblasts were obtained from patients recruited in Finland, after obtaining informed consent. The fibroblasts were expanded in fibroblast culture media containing Iscove’s Dulbecco’s modified Eagle’s medium (DMEM) (Thermo Fisher Scientific) with 20% fetal bovine serum, 1% Penicillin–Streptomycin, and 1% non-essential amino acids. Control and SZ human fibroblasts were reprogrammed with CytoTune-iPS 2.0 Sendai Reprogramming Kit (Thermo Fisher Scientific) according to the manufacturer’s instructions. Specifically, fibroblasts at 90% confluency were transduced with three separate vectors carrying genes *hOCT-3/4*, *hKLF-4*, *hSOX-2*, and *hc-MYC* to induce the pluripotency. The medium was changed 24 h after transduction and daily thereafter. At day 6, fibroblast culture medium was replaced with Essential 6 Medium (E6, Thermo Fisher Scientific) supplemented with 100 ng/ml basic fibroblast growth factor (bFGF). On the day after, the cells are re-plated onto six-well Matrigel-coated plates with a density of 60,000 cells/well. Between days 17–28, the individual colonies were picked up to 24-well Matrigel-coated plates containing Essential 8 Medium (E8, Thermo Fisher Scientific) and passaged with 0.5 mM EDTA weekly. Medium was changed every other day. The pluripotency of iPSCs was confirmed by the expression of pluripotent markers using immunocytochemistry (Oct-4, Sox2, TRA-1-81, SSEA4) and quantitative PCR (qPCR) (*OCT-4, SOX-2, NANOG,* and *LIN-28*). United Medix Laboratories Ltd in Helsinki (Finland) performed karyotyping analysis. The ability to form embryoid bodies (EBs) was confirmed by growing the hiPSCs in low-adherent plates for 2 weeks after which the EBs were plated down for the additional 2 weeks. The expression of proteins originating from the three germ layers was confirmed by immunocytochemistry (smooth muscle actin, BIIITubulin, and anti-alpha-fetoprotein).

### hiPSC differentiation to NPCs and neurons

Neural differentiation was performed according to Hicks et al.^40^ with minor modifications. Specifically, hiPSCs grown in E8 medium were incubated with10 µM SB431542 and 200 nM LDN-193189 (both from Selleckchem) for 10 days in neural differentiation medium (1:1 mix of DMEM/F12 and Neurobasal medium supplemented with 1% B27, 0.5% N2, 2 mM Glutamax, 50 IU/ml penicillin, and 50 μg/ml streptomycin (all from Thermo Fisher Scientific). After the induction, the visible rosettes containing differentiated neuroepithelial cells were detached from Matrigel-coated plates and transferred to non-adherent plates (Corning) in neural sphere medium (NSM—1:1 mix of DMEM/F12 and Neurobasal medium supplemented with 1% N2 supplement, 2 mM Glutamax, 50 IU/ml penicillin, and 50 μg/ml streptomycin (all from Thermo Fisher Scientific) supplemented with 25 ng/ml bFGF (Peprotech). The spheres were manually cut once a week, to maintain progenitor-state neural cell population, and the medium was then renewed every other day. For experimental purposes, 8–12 weeks old NPCs were dissociated with Accutase and plated in NSM medium supplemented with 20 ng/ml BDNF (Peprotech), 20 ng/ml GDNF (Peprotech), 1 mM dibutyryl-cyclicAMP (Merck), and 200 nM ascorbic acid (Merck) onto PORN/Matrigel-coated plates (with density 2–3 × 10^6^ cells/6 cm dish; 1 × 106 cells/6-well plate or 100,000 cell/24-well plate. The neurons were maintained 1 week before any experiments. Supplementary Table 7 summarizes the lines used in each experiment.

### Immunocytochemistry

Cells were fixed in 4% paraformaldehyde in phosphate-buffered saline (PBS) at room temperature (RT) for 20 min. hiPSCs and NPCs were permeabilized at RT for 1 h in 0.25% Triton X-100 in PBS. The unspecific binding sites were blocked in 5% normal goat serum in PBS at RT for 1 h. The following primary antibodies and dilutions were used: OCT-4 (Chemicon MAB4401), 1:400; NANOG (R&D Systems AF1997), 1:100; TRA-1-81 (Chemicon MAB4381), 1:200; SSEA4 (Chemicon MAB4304), 1:400; AFP (Sigma A8452), 1:500; SMA (Sigma A5228), 1:500; TUJ1 (Covance MMS-435P), 1:2000; MAP2 (Chemicon MAB3418), 1:200; VGLUT1 (Sigma V0389), 1:300; and GABA (Sigma 2052), 1:600. Secondary antibodies were Alexa goat 488 and 568 anti-rabbit (Invitrogen) and Alexa goat 488 and 568 anti-mouse (Invitrogen); all were used at 1:300. The nuclei were stained with 0.5 μg/ml DAPI (4’,6-diamidino-2-phenylindole, Sigma) and the coverslips were mounted with Vectashield or Fluoromount.

### Gene expression analysis

Gene expression analysis was performed with 2–3 months old hiPSC-derived cortical neurons. RNA was isolated from the plated neurons by mirVana kit (Thermo Fisher Scientific) according to the manufacturer’s protocol. The RNA quality was analyzed on the Agilent 2100 Bioanalyzer™ using an RNA6000 assay. Gene expression profile of five twin pairs discordant for schizophrenia and six control subjects were compared using whole transcriptome sequencing on the Illumina Hiseq 2500. The single-end sequencing reads were trimmed to remove adapters using cutadapt^41^ and the trimmed reads were aligned to human GRCh38 (hg38) genome assembly with Ensembl GRCh38.90 transcript annotations using STAR aligner, v. 2.5.2^42^ in quantification mode to get the gene-level read counts. Data normalization and differential expression analysis were performed using R package DESeq2, v. 1.16.1^43^. Schizophrenia status (ST, HT, or control) and sex (when applicable) were used as covariates in the DESeq2 model. In cases where the inter-twin comparisons were made, a paired analysis was used by including twin pair information in the model. For studying sex-specific differences in the schizophrenia status effect, an interaction term sex:schizophrenia status was tested. For the analysis of differential gene expression between males and females, only healthy control group samples were analysed by contrasting female samples with male samples. *P*-values were adjusted for multiple testing using the Benjamini–Hochberg multiple testing adjustment method^44^. Genes with absolute log2 fold change > 1 and adjusted *p* < 0.05 were considered as significantly differentially expressed in all comparisons. The primers used in qPCR were CHL1 = Hs00544069_m1, COL6A3 = Hs00915125_m1, DDX3Y = Hs00190539_m1, ETV1 = Hs00951951_m1, LHX1 = Hs00232144_m1, RPS4Y1 = Hs00606158_m1, SSTR2 = Hs00990356_m1, LIN28 = Hs00702808_s1, OCT4 = Hs00742896_s1, NANOG = Hs02387400_g1, and SOX2 = Hs01053049_s1. Differences between study groups were tested with one-way analysis of variance.

Hoffman et al.^45^ gene expression data were downloaded from Gene Expression Omnibus (accession GSE106589). Data from the two cell types, hiPSC-NPCs and hiPSC-neurons, were analysed separately using DESeq2, contrasting samples by diagnosis, COS (childhood-onset schizophrenia) vs. controls. Data were adjusted for covariates that were shown to have significant effects on data heterogeneity by Hoffman et al.^45^, i.e., fibroblast1 and fibroblast2 cell type composition scores and sex. Sex-specific differences in the COS gene expression signature was analysed as above, by testing the interaction term sex:COS in addition to the covariates listed above. DEGs between female and male within this dataset was analysed by contrasting healthy female controls and healthy male controls. *P*-value adjustment and significance filtering were carried out as described above.

The enrichment analyses were performed to DEGs. Biological Process Gene Ontology (GO BP) term and Kyoto Encyclopedia of Genes and Genomes (KEGG) pathway term over-representation analyses were performed using R package clusterProfiler. DEGs for the over-representation analysis were chosen using the adjusted *p*-value threshold of <0.05 and requiring at least twofold up- or downregulation in expression. The *p*-values of enrichment analysis were corrected for multiple testing using Benjamini–Hochberg multiple testing adjustment procedure. In addition, gene-set enrichment analysis (GSEA)^46^ of GO and KEGG terms using clusterProfiler was performed. GSEA uses all the analysed genes that are ordered based on the expression fold change, with genes having the largest statistically significant upregulation listed at the top and genes having the largest statistically significant downregulation at the bottom of the list. The ordering was done based on the classic GSEA signed ranking score that was calculated for each gene by multiplying the log2 fold change by −log10 (adjusted *p*-value). GSEA then calculated a running-sum statistic, enrichment score (ES), while walking down the ranked gene list, generating positive ES if a gene set was enriched at the top of the list or negative ES if the gene set was enriched at the bottom of the list. ES was further normalized to normalized ES accounting for differences in gene-set sizes and in correlations between gene sets and the expression dataset.

### Proteomics

The pelleted hiPSC-derived neurons were lysed in buffer containing 8 M urea, 75 mM NaCl, and 50 mM Tris with pH adjusted to 8.2. The lysates were sonicated with microtip at 80% amplitude and 1 cycle twice for 10 s (UP50H Hielscher), and centrifuged at 12,500 × *g* for 10 min to eliminate non-lysed tissue. The protein content was determined by Bradford assay. The samples were processed through the SysQuant® workflow using Tandem Mass Tag (TMT®) reagents within two TMT® 10plexes. A reference pool containing all samples was also included in both TMT® 10plexes. Batch effects in peptide, phosphopeptide, and protein data were removed using the removeBatchEffect function of the limma R package and the successfulness of the batch removal was inspected by generating principal component analysis (PCA) plots. Differential protein expression was analyzed using limma. Pairedness of the twin samples was taken into account by using the limma function duplicateCorrelation. *P*-values were adjusted for multiple testing using the Benjamini–Hochberg multiple testing adjustment method. Proteins with *p* < 0.05 and at least 30% increase or decrease in expression (log2-fold change > 0.379) were considered differentially expressed. The adjusted *p*-values are included in results tables. PCA plots were generated after feature selection, using differentially expressed features from the comparison of ST twins and unaffected (HT) twins with statistical significance levels of *p* < 0.01 for peptides and phosphopeptides, and *p* < 0.05 for protein data. GSEA analysis^46^ of GO BP terms and KEGG pathways was performed similarly as for RNA-Sequencing data. GSEA analysis does not rely on any defined threshold for differential expression and can thus detect situations where proteins associated with a given pathway term have changed in a small but coordinated way.

Thresholding protein differences at *p* < 0.01 yielded 42 proteins, of which 37 had a corresponding gene. Testing gene-level differences of the genes whose corresponding protein was different between ST and unaffected siblings yielded only one significant gene (COL6A3) when the threshold was *p* = 0.05, Bonferroni corrected (see Supplementary Data 31).

### CNV analysis

Genomic DNA was extracted using Qiagen’s DNeasy Blood and Tissue kit according to the manufacturer’s instructions. Briefly, fibroblasts were lysed in buffer containing proteinase K and RNase A for 10 min at 56 °C. DNA was precipitated with 100% ethanol and then washed three times with buffer containing ethanol prior to elution. Genome scans were performed using Agilent Microarray Kit in Functional Genomics Unit (Biomedicum, Helsinki). Genomic DNA (500 ng) was digested with AluI and RsaI enzymes. Digested gDNA samples were labeled using random primers, fluorescent-labeled dUTP nucleotides (Cy3 and Cy5), and the exo-Klenow fragment. Experimental samples were labeled with Cy5 and reference samples with Cy3. Commercial genomic DNA from Agilent was used as a reference according to gender-informed by the customer. Combined experimental and reference samples were mixed with Human Cot-1 DNA, blocking agent, and hybridization buffer, and hybridized to Agilent Human Genome CGH 4 × 180 K SurePrint G3 Microarrays for 40 h at 67 °C. Microarrays were scanned with Agilent Scanner G2505C, using manufacturer-provided protocol. Feature Extraction software was used for image analysis. The data on each chip was compared against reference genome hg19 and analyzed with 2 kb window size. A minimum of three sequential amplified, deleted, or gained probes was used to filter possible copy number variants (CNVs). The lists of concordant, overlapping, and de novo mutations between the twins in each pair were then listed (see Supplementary Table 6).

### Electrophysiology

To preliminary test functional expression of glutamate and GABA receptors in neuronal cultures, we first used the whole cell patch-clamp recordings using EPC10 amplifier and PatchMaster software (HEKA electronics, Germany). Experiments were performed at RT (21-23oC). Cells were held in voltage-clamp mode at −70 mV continuously perfused with the extracellular basic solution (BS) contained (in mM): 152 NaCl, 2.5 KCl, 2 CaCl_2_, 10 HEPES, 10 d-Glucose pH 7.4 adjusted with NaOH. Intracellular solution contained: 130 CsCl, 5 MgCl_2_, 10 HEPES, 5 EGTA, 0.5 CaCl_2_, 2 Mg-ATP, 0.5 Na-GTP, 5 KCl with pH 7.2 adjusted by CsOH. Patch pipets had a resistance 4–5 MOhm. The agonists GABA (100 μM) or glutamate (100 μM together with 10 μM Glycine) were applied for 2 s via the fast local perfusion system (RSC-200, BioLogic, France).

### Calcium imaging

Calcium imaging was performed with 2–3 months old hiPSC-derived cortical-like neurons. The neurons were plated onto PORN/Matrigel-coated circular coverslips (9 mm diameter) in 48-well plate at a density of 50,000 cells/well for 1 week in NSM medium supplemented with maturation factors. Clozapine (15 µM, Sigma) or dimethyl sulfoxide was added for three additional days before measurement (see Supplementary Table 2).

To quantify and compare the functional expression of glutamate and GABA receptors in neuronal cultures, we used calcium-imaging technique as previously described^47^. Briefly, neuronal cultures were loaded with the cell-permeable indicator Fluo-4am (F10471, Life Technologies, USA) for 30 min at 37 °C, followed by short washout, and placed in the perfusion chamber mounted on the stage of Olympus IX7010 microscope. Neurons were continuously perfused by BS. Glutamate (100 µM with the co-agonist glycine 10 µM in magnesium-free solution promoting activation of NMDA receptors subtype) or GABA (100 µM) were applied for 2 s by a fast perfusion system (RSC-200). KCl (30 mM) application for 2 s was used to distinguish excitable neurons from possible non-neuronal cells. Calcium ionophore ionomycin (10 µM) was applied for 2 s at the end of each recording and this calcium transient was used for normalization of receptor responses. Fluorescence was detected with the Till Photonics imaging system (FEI GmbH, Munich, Germany) equipped with a 12 bit CCD Camera (SensiCam, Germany) with a light excitation wavelength of 494 nm and adequate filters.

### Statistical analysis of calcium imaging

Calcium responses to neurotransmitters were evaluated from changes in fluorescence intensity of individual neurons. To this end, regions of interest (ROI) of round shape around the cell body were applied to whole image containing up to 77 neurons with TILLvision Imaging Software (TILL Photonics GmbH, Gräfelfing, Germany). To distinguish from non-neuronal cells, ROI was taken at the time point corresponding to KCl-induced activation of excitable cells (neurons). The intensity values in each ROI were averaged at each time point to form a fluorescence signal for each neuron. Baseline levels of fluorescence were obtained by averaging signals from three cell-free regions. For the analysis, first, we subtracted the background signal (average of the three background ROIs) from the fluorescence time series. Second, we de-noised the background-subtracted ROI-wise fluorescence time series. To this end, we used wavelet de-noising, and, in particular, the maximal overlap discrete wavelet transform (also known as stationary wavelet transform) based on level 3 Haar wavelets^48^. The wavelet coefficients were soft-thresholded with Donoho and Johnstone’s^49^ universal threshold with level-dependent re-scaling as implemented in the Matlab function wden (Mathworks, Inc. Natick, MA, US). This de-noising effectively removed noise, while retaining the important characteristics of the fluorescence signal. The calcium response to neurotransmitters was quantified as dF* = (*f* − *f*0)/*f*0, where *f* is the maximum of the de-noised fluorescence time series during 20 s window following the application of KCl, GABA, or Glutamate + Glycine (either with or without Mg) and *f*0 is the baseline extracted as the minimum of the de-noised fluorescence time series in a window 50 s before the application of the neurotransmitters. The calcium responses were calibrated by dividing them by dF* corresponding to ionomycin. The application of signal de-noising made it possible to use minimum and maximum in the estimates without making the process excessively sensitive to noise. Finally, GABA and Glutamate + Glycine responses were still calibrated by dividing them by the KCl response.

We tested whether the calcium response to the neurotransmitters of the ST subjects differed from their healthy twins on average. For this, we used random-effects hierarchical model based on sufficient summary statistic approach^50^. The first level of the analysis pooled all the cells of the single subject, before and after the treatment, and estimated the subject and treatment-wise means and variances using the standard unbiased estimates. Outliers were removed before the computation of the summary statistics. A measurement was considered as an outlier if its value was more than three times consistency-corrected median absolute distance from the median, which is a standard procedure. The second level was based on the inverse variance-weighted random-effects model, where the between-subject variance was estimated with the method of DerSimonian and Laird. For testing the difference of the treatment we estimated the variance of the difference between ST and unaffected twin by summing the variances from the first level. The researcher was blind to the genotype of the tested culture.

All the analysis codes were written in Matlab and are available at https://github.com/jussitohka/CalciumImaging.

### Reporting summary

Further information on research design is available in the Nature Research Reporting Summary linked to this article.

## Supplementary information

Supplementary Information

Peer Review

Reporting Summary

Description of Additional Supplementary Files

Supplementary Data 1

Supplementary Data 2

Supplementary Data 3

Supplementary Data 4

Supplementary Data 5

Supplementary Data 6

Supplementary Data 7

Supplementary Data 8

Supplementary Data 9

Supplementary Data 10

Supplementary Data 11

Supplementary Data 12

Supplementary Data 13

Supplementary Data 14

Supplementary Data 15

Supplementary Data 16

Supplementary Data 17

Supplementary Data 18

Supplementary Data 19

Supplementary Data 20

Supplementary Data 21

Supplementary Data 22

Supplementary Data 23

Supplementary Data 24

Supplementary Data 25

Supplementary Data 26

Supplementary Data 27

Supplementary Data 28

Supplementary Data 29

Supplementary Data 30

Supplementary Data 31

Supplementary Data 32

## Data Availability

RNA-seq and proteomic raw data are shown on individual level in the Supplementary data 7, 8, 9, 10, 29, 30, and 32. All other relevant data are available on request from the authors.
