# Supplementary material for: Sex-specific transcriptional and proteomic signatures in schizophrenia
Source: Nat Commun. 2019 Sep 2;10:3933. doi: 10.1038/s41467-019-11797-3 (PMC6718673; doi:10.1038/s41467-019-11797-3)
Supplement: Supplementary file 1 — Supplementary Information [file 41467_2019_11797_MOESM1_ESM.docx]

**Supplementary Information**

**Sex-specific transcriptional and proteomic signatures in schizophrenia**

Jari Tiihonen, Marja Koskuvi, Markus Storvik, Ida Hyötyläinen, Yanyan Gao, Katja A. Puttonen, Raisa Giniatullina, Ekaterina Poguzhelskaya, Ilkka Ojansuu, Olli Vaurio, Tyrone D. Cannon, Jouko Lönnqvist, Sebastian Therman, Jaana Suvisaari, Jaakko Kaprio, Lesley Cheng, Andrew F. Hill, Markku Lähteenvuo, Jussi Tohka, Rashid Giniatullin, Šárka Lehtonen, Jari Koistinaho

**Supplementary Table 1.** Summary of patients and hiPS lines used in this study.

**Supplementary Table 2.** Neuronal calcium imaging of affected and unaffected twins.

**Supplementary Table 3.** Genomic location counts of the observed *de novo* CNVs, partially overlapping CNVs between the twins (counted as one), and concordant CNVs observed in both twins (counted as one).

**Supplementary Table 4.** Pathways, biological processes, cell compartments, and molecular functions associated with genes on any CNVs observed in the study.

**Supplementary Table 5.** Pathways, biological processes, cell compartments, and molecular functions associated with genes on *de novo* CNVs observed in at least one twin.

**Supplementary Table 6.** Enriched transcription factor binding sites predicted to all genes associated with the genomic range of any observed CNVs.

**Supplementary Table 7.** Summary of lines included in each comparison set.

**Supplementary Table 8.** Tiihonen and Hoffman DEGs comparisons summary

**Supplementary Table 9.** Proportions of sex-specific genes in Tiihonen et al. and Hoffman et al. data sets.

**Supplementary Table 10.** DEGs from SZ: Sex interaction test.

**Supplementary Table 11.** RNA-SEQ Read Alignment.

**Supplementary Figure 1.** Characterization of control hiPSC lines.

**Supplementary Figure 2.** Characterization of female monozygotic twin hiPSC lines.

**Supplementary Figure 3.** Immunocytochemical analysis of the embryoid body (EB) formation in female monozygotic twins and healthy female controls.

**Supplementary Figure 4.** Characterization of male monozygotic twin hiPSC lines.

**Supplementary Figure 5.** Overlap of sex-specific genes (comparison of healthy males vs. females) in three different datasets.

**Supplementary Table 1. Summary of patients and hiPS lines used in this study.**

| ***Abbreviations of hiPSC lines*** | ***Group*** | ***Age at biopsy (years)*** | ***Sex*** | ***Medication*** | ***PANSS positive*** | ***PANSS negative*** | ***PANSS general*** | ***PANSS total*** |
| --- | --- | --- | --- | --- | --- | --- | --- | --- |
| SZ1 (Pair 1) | Unaffected twin | 47 | Female | - | 10 | 13 | 26 | 49 |
| SZ2 | Affected twin | 47 | Female | Clozapine | 27 | 32 | 54 | 113 |
| SZ3 (Pair 2) | Unaffected twin | 68 | Female | - | 7 | 7 | 18 | 32 |
| SZ4 | Affected twin | 68 | Female | - | 7 | 7 | 20 | 34 |
| SZ5 (Pair 3) | Affected twin | 66 | Male | Zuclopenthixol | 18 | 21 | 38 | 77 |
| SZ6 | Unaffected twin | 66 | Male | - | 7 | 7 | 17 | 31 |
| SZ7 (Pair 4) | Affected twin^1^ | 69 | Female | Previously clozapine, now sertindole and quetiapine | 25 | 24 | 50 | 99 |
| SZ8 | Unaffected twin^1,2^ | 69 | Female | - | 7 | 7 | 21 | 35 |
| SZ9 (Pair 5) | Unaffected twin | 45 | Female | - | 7 | 7 | 17 | 31 |
| SZ10 | Affected twin | 45 | Female | Clozapine | 14 | 16 | 23 | 53 |
| SZ11 (Control 1) | Control | 44 | Male | - | 7 | 7 | 16 | 30 |
| SZ12 (Control 2) | Control | 59 | Female | - | 7 | 7 | 16 | 30 |
| SZ13 (Control 3) | Control | 49 | Female | - | 7 | 7 | 16 | 30 |
| SZ14 (Control 4) | Control | 64 | Female | - | 7 | 7 | 16 | 30 |
| SZ15 (Pair 6) | Affected twin^3^ | 40 | Male | Olanzapine and quetiapine | 13 | 14 | 30 | 57 |
| SZ16 | Unaffected twin^3^ | 40 | Male | - | 7 | 7 | 16 | 30 |
| SZ17 (Control 5) | Control | 63 | Male | - | 7 | 7 | 16 | 30 |
| SZ18 (Control 6) | Control | 50 | Female | - | 7 | 7 | 16 | 30 |

PANSS, positive and negative syndrome scale

Pair 2 was excluded from the study based on PANSS score of the index twin that did not differ either from unaffected twin or healthy controls. S27 had to discontinue clozapine due to side effects.

^1^ Father diagnosed with schizophrenia.

^2^ Son diagnosed with schizophrenia.

^3^ Uncle diagnosed with schizophrenia.

**Supplementary Table 2. Neuronal calcium imaging of affected and unaffected twins.**


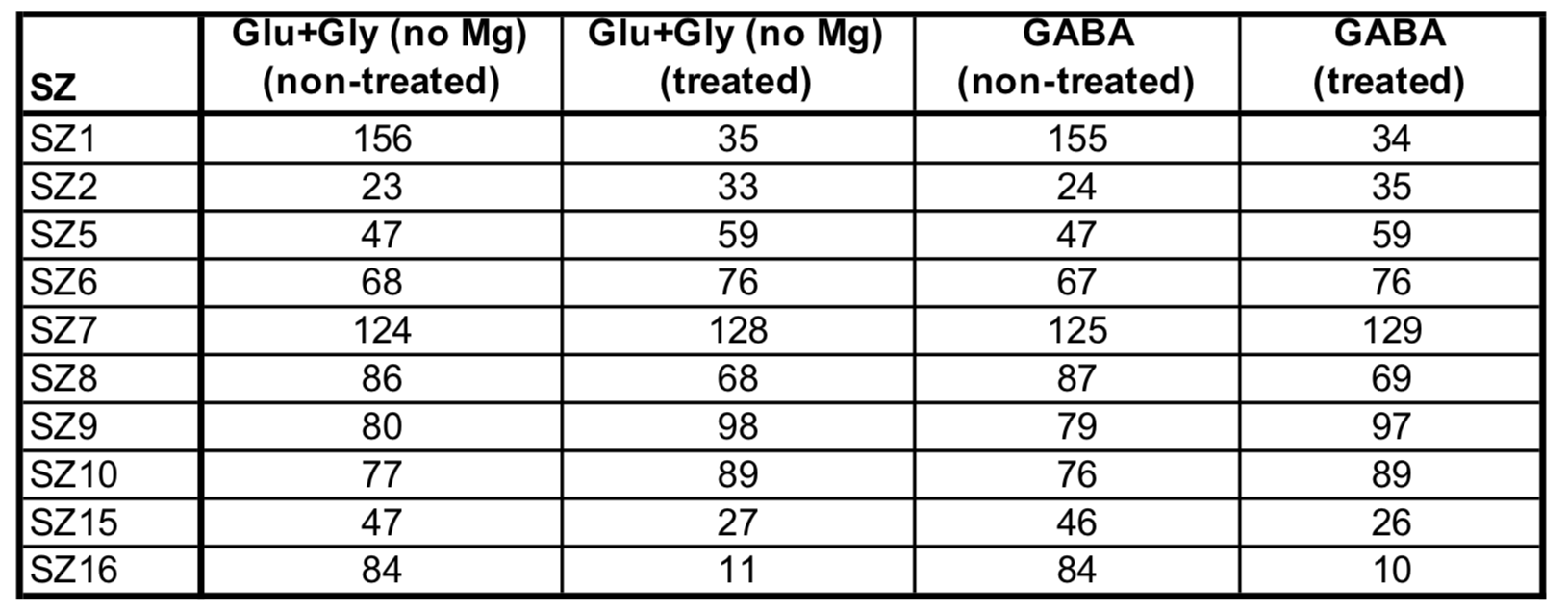


The number of measured neurons from each subject in different treatment conditions.

**Supplementary Table 3. Genomic location counts of the observed *de novo* CNVs, partially overlapping CNVs between the twins (counted as one), and concordant CNVs observed in both twins (counted as one).**

| **Chromosome** | **concordant** | ***de novo* unaffected** | **Overlapping** | ***de novo* affected** | **summary** |
| --- | --- | --- | --- | --- | --- |
| chr1 | 4 | 1 |  | **2** | **7** |
| chr2 | 10 |  | 2 | **1** | **13** |
| chr3 | 9 |  | 2 | **2** | **13** |
| chr4 | 4 |  |  | **1** | **5** |
| chr5 |  | 1 |  |  | **1** |
| chr6 | 5 |  |  |  | **5** |
| chr7 | 4 |  |  |  | **4** |
| chr8 | 12 | 8 |  | **1** | **21** |
| chr9 | 1 |  |  | **2** | **3** |
| chr10 | 1 | 2 | 2 | **4** | **9** |
| chr11 | 2 |  |  |  | **2** |
| chr12 | 2 |  |  |  | **2** |
| chr13 |  |  |  | **1** | **1** |
| chr14 | 21 | 2 | 3 | **6** | **32** |
| chr15 | 4 |  |  |  | **4** |
| chr16 | 4 |  | 1 | **2** | **7** |
| chr17 | 3 | 1 | 1 |  | **5** |
| chr18 |  |  |  |  | **0** |
| chr19 |  |  |  |  | **0** |
| chr20 |  |  |  |  | **0** |
| chr21 |  | 1 |  |  | **1** |
| chr22 | 13 |  | 1 | **2** | **16** |
| chrX | 3 |  |  | **1** | **4** |
| chrY | 5 | 2 | 1 | **1** | **9** |
| **total:** | **107** | **18** | **13** | **26** | **164** |

**Supplementary Table 4. Pathways, biological processes, cell compartments, and molecular functions associated with genes on any CNVs observed in the study.**

| **Enriched KEGG pathways** | **number of genes** | **p-value** |
| --- | --- | --- |
| Olfactory transduction a) | 15 | 2.6E-5 |
| Nitrogen metabolism | 4 | 5.6E-4 |
| Chemical carcinogenesis | 5 | 7.7E-3 |
| Drug metabolism - other enzymes | 4 | 1.0E-2 |
| Collecting duct acid secretion | 3 | 2.9E-2 |
| Caffeine metabolism | 2 | 4.8E-2 |
| Hematopoietic cell lineage | 4 | 5.1E-2 |
| Rheumatoid arthritis | 4 | 5.5E-2 |
| Synaptic vesicle cycle | 3 | 1.3E-1 |
| Drug metabolism - cytochrome P450 | 3 | 1.4E-1 |
| **Enriched biological processes (GO-terms)** | **number of genes** | **p-value** |
| gonadal mesoderm development | 7 | 5.7E-11 |
| spermatogenesis | 24 | 9.4E-11 |
| defense response to bacterium | 13 | 1.1E-7 |
| detection of chemical stimulus involved in sensory perception | 11 | 1.3E-7 |
| detection of chemical stimulus involved in sensory perception of smell | 17 | 3.1E-5 |
| G-protein coupled receptor signaling pathway | 24 | 2.5E-4 |
| innate immune response | 13 | 4.0E-3 |
| single fertilization | 5 | 4.6E-3 |
| one-carbon metabolic process | 4 | 4.8E-3 |
| histone acetylation | 4 | 6.8E-3 |
| **Enriched cell compartments (GO-terms)** | **number of genes** | **p-value** |
| extracellular region | 32 | 3.4E-3 |
| cell surface | 13 | 2.4E-2 |
| Golgi lumen | 5 | 2.5E-2 |
| late endosome membrane | 4 | 1.1E-1 |
| plasma membrane | 56 | 1.3E-1 |
| microvillus | 3 | 1.4E-1 |
| phagocytic vesicle membrane | 3 | 1.5E-1 |
| intracellular membrane-bounded organelle | 10 | 2.0E-1 |
| integral component of plasma membrane | 21 | 2.1E-1 |
| apical part of cell | 3 | 2.2E-1 |
| **Enriched molecular functions (GO-terms)** | **number of genes** | **p-value** |
| olfactory receptor activity | 17 | 6.5E-6 |
| transmembrane signaling receptor activity | 11 | 5.7E-5 |
| G-protein coupled receptor activity | 19 | 2.6E-4 |
| HECT domain binding | 3 | 2.9E-4 |
| carbonate dehydratase activity | 4 | 3.3E-4 |
| nucleotide binding | 12 | 7.4E-4 |
| L-amino acid transmembrane transporter activity | 3 | 7.1E-3 |
| histone acetyltransferase activity | 4 | 1.2E-2 |
| arylamine N-acetyltransferase activity | 2 | 3.0E-2 |
| CCR6 chemokine receptor binding | 2 | 3.9E-2 |

GO-term: gene ontology term; KEGG: Kyoto encyclopedia of genes; n of genes: number of genes associated with the named; P-value: modified Fisher Exact P-Value. Footnotes: a) Olfactory transduction pathway genes: OR11H12, OR2T10, OR2T11, OR2T34, OR4C6, OR4K1, OR4K2, OR4K5, OR4M1, OR4M2, OR4N2, OR4N4, OR4P4, OR4Q3, OR4S2.

When calculating all CNV in all pairs, 107 of all 164 detected CNVs were concordant and observed as identical in both twins in at least one pair. Among all CNVs corresponding to 404 genes, the thematic analysis suggested enrichment in GO cell compartment terms “extracellular region” and “cell surface”, and GO molecular function term “olfactory receptor activity” (17 genes, p=0.000065), mostly being members of olfactory receptor family 2 and 4. Also, xenobiotic metabolism genes such as NAT1, NAT2, UGT2B17, CYP2E1, and GSTT1 were observed among the genes on all CNVs. However, the putative discrepancy between the Finnish population and the reference genome cannot be estimated reliably. As indicated by transcription factor binding site analysis of all 404 genes on all CNVs using X2K method including JASPAR and TRANSFAC databases, neuronal transmission related GRID2 (loss CNV) and NPY4R (gain CNV) were also among the 8 genes regulated by SMAD4 transcription factor binding site, the p-value for enrichment for SMAD4 containing genes was 0.04. SMAD4 itself has been reported to be upregulated in schizophrenia, suggesting a putative site for genome-environment interactions. Also, the most enriched transcription factor binding site was androgen receptor binding site (p=0.005) with 15 putative targets, suggesting still another site for genome-environment interactions. Those genes included SLC18A1 that is also present in the enriched “synaptic vesicle cycle” pathway among *de novo* CNVs in non-affected twins only (SLC18A1 gain CNV).

The *de novo* CNVs in twins have genomic background effect, but unfortunately, the number of such CNVs were low. The 26 *de novo* CNVs in affected twins but not in non-affected twins only were located on areas corresponding to 84 genes, and the 18 *de novo* mutations on unaffected twin only corresponding to 213 genes. Among the 26 de novo mutations in affected twin only, there was one enriched KEGG pathway, “neuroactive ligand-receptor interaction” (p=0.048) corresponding to glutamatergic GRID2 and neuropeptide Y receptor NPY4R, also represented by the CH17-360D5.1 variant in the list. When compared to transcriptomic data, there were no significant mRNA expression differences between the pairs in case of GRID2 or NPY4R in the tested model, despite the well-established involvement of glutamatergic system in schizophrenia.

**Supplementary Table 5. Pathways, biological processes, cell compartments, and molecular functions associated with genes on *de novo* CNVs observed in at least one twin.**

| **among affected twin *de novo* CNVs only** | |  |
| --- | --- | --- |
| **Enriched KEGG pathways** | **number of genes** | **p-value** |
| Neuroactive ligand-receptor interaction a) | 3 | 4.8E-2 |
| Ribosome biogenesis in eukaryotes | 1 | 1.0E0 |
| Herpes simplex infection | 1 | 1.0E0 |
| RNA transport | 1 | 1.0E0 |
| Staphylococcus aureus infection | 1 | 1.0E0 |
| Long-term depression | 1 | 1.0E0 |
| Regulation of actin cytoskeleton | 1 | 1.0E0 |
| Hematopoietic cell lineage | 1 | 1.0E0 |
| Transcriptional misregulation in cancer | 1 | 1.0E0 |
| Pertussis | 1 | 1.0E0 |
|  |  |  |
| **among unaffected twin *de novo* CNVs only** | |  |
| **Enriched KEGG pathways** | **number of genes** | **p-value** |
| Nitrogen metabolism | 4 | 9.9E-5 |
| Collecting duct acid secretion | 3 | 9.5E-3 |
| Drug metabolism - other enzymes | 3 | 2.6E-2 |
| Caffeine metabolism | 2 | 2.7E-2 |
| Synaptic vesicle cycle b) | 3 | 4.7E-2 |
| Rheumatoid arthritis | 3 | 8.4E-2 |
| Phagosome | 3 | 2.1E-1 |
| Metabolic pathways c) | 10 | 2.2E-1 |
| Vibrio cholerae infection | 2 | 2.5E-1 |
| Epithelial cell signaling in Helicobacter pylori infection | 2 | 3.1E-1 |

GO-term: gene ontology term; KEGG: Kyoto encyclopedia of genes; n of genes: number of genes associated with the named; P-value: modified Fisher Exact P-Value. Footnotes: a) genes in “Neuroactive ligand-receptor interaction”: GRID2, NPY4R, CH17-360D5.1 b) genes in “Synaptic vesicle cycle”: ATP6V0D2, ATP6V1B2, SLC18A1 c) genes in “Metabolic pathways”: ATP6V0D2, ATP6V1B2, NAT1, NAT2, ASAH1, ACOT1, CSGALNACT1, DPYS, MTMR7, TUSC3.

**Supplementary Table 6. Enriched transcription factor binding sites predicted to all genes associated with the genomic range of any observed CNVs:**

| **Rank** | **Transcription Factor** | **Hypergeometric p-value** | **List of TF targets on full lists of CNVs** |
| --- | --- | --- | --- |
| 1 | **AR** | 0.005369 | CNBD1 **SLC18A1** MMP16 COL22A1 COL26A1 CNTN4 TBC1D5 MGMT TUSC3 CSGALNACT1 NLGN4Y KBTBD11 PDZD2 LRP12 DOCK1 |
| 2 | NFE2L2 | 0.007152 | FAM135B CSMD3 KCNV1 CA2 GOLPH3 RBMY1A1 RSPO2 MGMT ANGPT1 OXR1 ZFPM2 EMC2 LPL LRP12 |
| 3 | SUZ12 | 0.02581 | DLGAP2 MMP16 SLC7A2 GRID2 COL26A1 KCNV1 FGF20 CNTN4 CSGALNACT1 LPL CSMD1 PTPRE FOXI2 CD24 RSPO2 EBF3 ZFPM2 RALYL |
| 4 | STAT3 | 0.03056 | KIAA1456 SKAP2 TDRP DLC1 |
| 5 | **SMAD4** | 0.03925 | BAGE **GRID2** ANGPT1 FOXI2 ABRA MSR1 DLC1 **NPY4R** |
| 6 | RFX5 | 0.07617 | HLA-DRB5 EBAG9 HLA-H INTS10 EMC2 ZFAND4 CA13 |
| 7 | REST | 0.07650 | CYP2E1 DLGAP2 USP9Y LINGO2 FOXI2 ZDHHC2 DDX3Y CA3 GLRX3 ATP6V1B2 RBMY1A1 TUSC3 PSD3 |
| 8 | SOX2 | 0.1378 | SKAP2 DPYS CD24 ACOT1 ASAH1 MTUS1 EBF3 CA13 |
| 9 | EGR1 | 0.1547 | PCM1 SLC18A1 EMC2 ZFAND4 |
| 10 | ZEB1 | 0.1562 | LINC00273 GOLPH3 |

**Supplementary Table 7. Summary of lines included in each comparison set.**

**
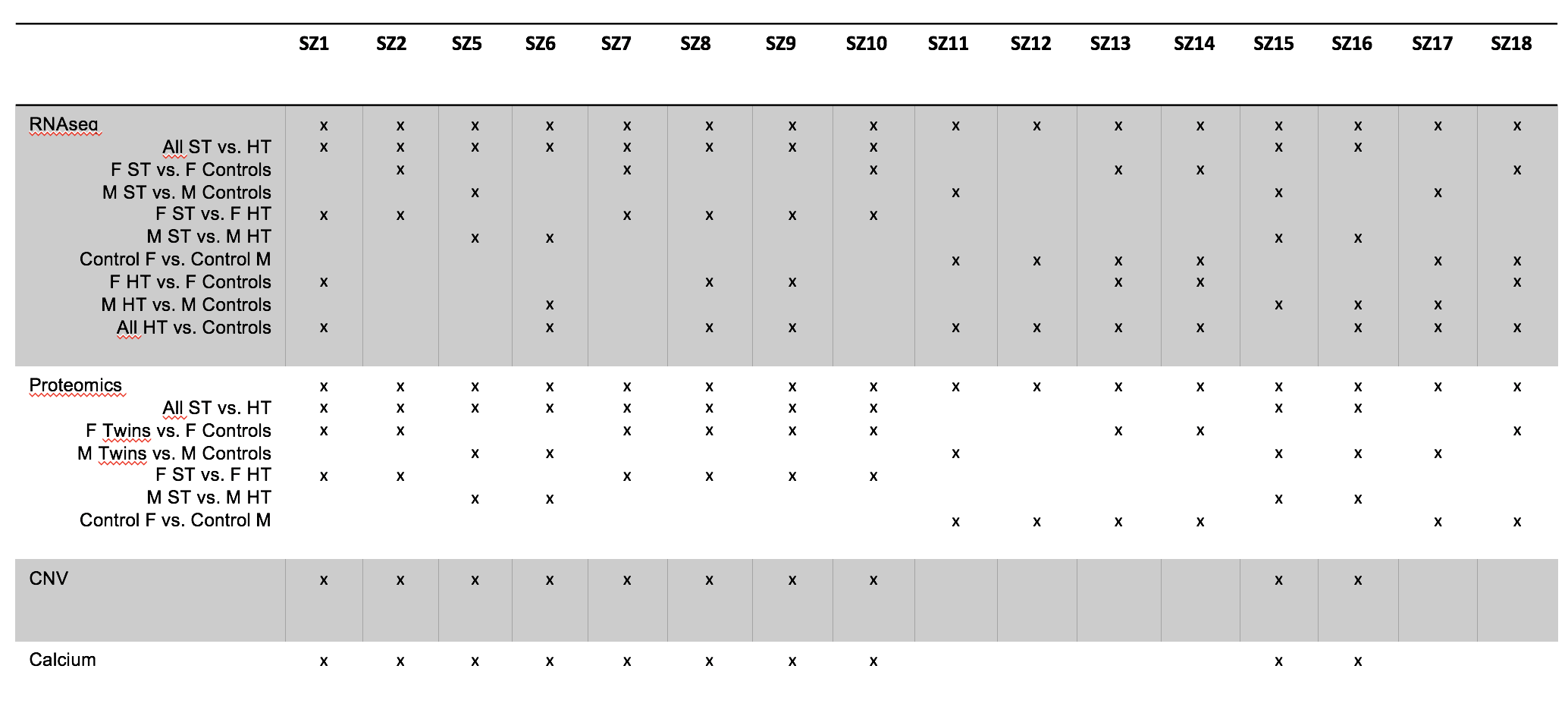
**

ST, affected twin; HT, unaffected twin; F, female; M, male

**Supplementary Table 8.** Comparison of DEGs in Tiihonen et al. and Hoffman et al. datasets.

|  | **Tiihonen data** | | **Hoffman data** |  |  |  |  |  |  |
| --- | --- | --- | --- | --- | --- | --- | --- | --- | --- |
| **Comparison** | **Comparison name** | **No of DEGs** |  | **No of DEGs in NPCs** |  | **No of DEGs in Neurons** |  |  |  |
| **number** | **(all comparisons without pair 2 samples)** | **(Adjusted p-value < 0.05 & abs(Log2 foldchange) > 1)** | **Comparison name** | **(Adjusted p-value < 0.05 & abs(Log2 foldchange) > 1)** | **DEGs overlapping with Tiihonen data** | **(Adjusted p-value < 0.05 & abs(Log2 foldchange) > 1)** | **DEGs overlapping with Tiihonen data** | **DEGs in both Hoffman cell types** |  |
| **21** | All HT (5) vs Controls (5) | 355 | Not determined as no healthy twin patients in Hoffman data | | | | | | |
| **24** | All ST (5) vs Controls (5) | 232 | All COS(23) vs Controls (24) (sex included in the model) | 92 | 2 | 60 | 2 | 18 |  |
| **25** | Female ST (3) vs Female Controls (4) | 306 | Female COS (10) vs Female Controls (9) | 129 | 2 | 127 | 2 | 23 |  |
| **26** | Male ST (2) vs Male Controls (2) | 342 | Male COS (13) vs Male Controls (15) | 82 | 6 | 78 | 1 | 23 |  |
| **16** | Female Controls (4) vs Male Controls (2) | 2327 | Female Controls (9) vs Male Controls (15) | 114 | 6 | 244 | 18 | 61 |  |
|  |  |  |  |  |  |  |  |  |  |
| **Analysis using SZ:sex interaction term** | |  |  |  |  |  |  |  |  |
| **21** | All HT (5) vs Controls (5), without control 2 | 538 |  |  |  |  |  |  |  |
| **24** | All ST (5) vs Controls (5), without control 2 | 99 | All COS(23) vs Controls (24) | 78 | 2 | 145 | 1 | 8 |  |

**Supplementary Table 9.** Proportions of sex-specific genes in Tiihonen et al. and Hoffman et al. datasets.

| **Chi-squared tests of dependency** | |  | |  | |  | |  |
| --- | --- | --- | --- | --- | --- | --- | --- | --- |
|  |  | |  | |  | |  | |
|  |  | |  | |  | |  | |
|  | **Tiihonen data (with sex covariate in the model):** | | | |  | |  | |
|  |  | |  | |  | |  | |
| **Comparison number** |  | | **Genes in comparison female ctrl vs male ctrl** | | **Differentially expressed genes in comparison HT vs ctrl (sex in the model)** | |  | |
| **21** | **Differentially expressed in female vs male** | | 2327 | | 59 | | **Pearson’s Chi-squared test:** | |
|  | **Not differentially expressed in female vs male** | | 17135 | | 296 | | **p-value (computed from asymptotic chi-squared distribution of the test statistic with Yate’s continuity correction) = 0.009508535** | |
|  |  | |  | |  | |  | |
|  |  | |  | |  | |  | |
|  |  | | **Genes in comparison female ctrl vs male ctrl** | | **Differentially expressed genes in comparison SZ vs ctrl (sex in the model)** | |  | |
| **24** | **Differentially expressed in female vs male** | | 2327 | | 70 | | **Pearson’s Chi-squared test:** | |
|  | **Not differentially expressed in female vs male** | | 17 135 | | 162 | | **p-value (computed from asymptotic chi-squared distribution of the test statistic with Yate’s continuity correction) = 7.754166e-17** | |
|  |  | |  | |  | |  | |
|  |  | |  | |  | |  | |
|  | **Hoffman et al. Data (two cell types analysed separately):** | | | |  | |  | |
|  |  | |  | |  | |  | |
|  | **hiPSC-derived 6-week-old forebrain neurons:** | |  | |  | |  | |
|  |  | | **Genes in comparison female ctrl vs male ctrl** | | **Differentially expressed genes in comparison SZ vs ctrl** | |  | |
| **24 neuron** | **Differentially expressed in female vs male** | | 244 | | 3 | | **Pearson’s Chi-squared test:** | |
|  | **Not differentially expressed in female vs male** | | 39 571 | | 57 | | **p-value (computed from asymptotic chi-squared distribution of the test statistic with Yate’s continuity correction) = 0.0004572278** | |
|  |  | |  | |  | |  | |
|  |  | |  | |  | |  | |
|  |  | | **Genes in comparison female ctrl vs male ctrl** | | **Differentially expressed genes in comparison female SZ vs female ctrl** | |  | |
| **25 neuron** | **Differentially expressed in female vs male** | | 244 | | 13 | | **Pearson’s Chi-squared test:** | |
|  | **Not differentially expressed in female vs male** | | 39 571 | | 114 | | **p-value (computed from asymptotic chi-squared distribution of the test statistic with Yate’s continuity correction) = 1.461758e-38** | |
|  |  | |  | |  | |  | |
|  |  | |  | |  | |  | |
|  |  | | **Genes in comparison female ctrl vs male ctrl** | | **Differentially expressed genes in comparison male SZ vs male ctrl** | |  | |
| **26 neuron** | **Differentially expressed in female vs male** | | 244 | | 17 | | **Pearson’s Chi-squared test:** | |
|  | **Not differentially expressed in female vs male** | | 39 571 | | 61 | | **p-value (computed from asymptotic chi-squared distribution of the test statistic with Yate’s continuity correction) = 6.708661e-112** | |
|  |  | |  | |  | |  | |
|  |  | |  | |  | |  | |
|  | **hiPSC-derived neural progenitor (NPC) cells:** | |  | |  | |  | |
|  |  | | **Genes in comparison female ctrl vs male ctrl** | | **Differentially expressed genes in comparison SZ vs ctrl** | |  | |
| **24 NPC** | **Differentially expressed in female vs male** | | 114 | | 3 | | **Pearson’s Chi-squared test:** | |
|  | **Not differentially expressed in female vs male** | | 36 689 | | 89 | | **p-value (computed from asymptotic chi-squared distribution of the test statistic with Yate’s continuity correction) = 4.132552e-05** | |
|  |  | |  | |  | |  | |
|  |  | |  | |  | |  | |
|  |  | | **Genes in comparison female ctrl vs male ctrl** | | **Differentially expressed genes in comparison female SZ vs female ctrl** | |  | |
| **25 NPC** | **Differentially expressed in female vs male** | | 114 | | 13 | | **Pearson’s Chi-squared test:** | |
|  | **Not differentially expressed in female vs male** | | 36 689 | | 116 | | **p-value (computed from asymptotic chi-squared distribution of the test statistic with Yate’s continuity correction) = 9.809623e-74** | |
|  |  | |  | |  | |  | |
|  |  | |  | |  | |  | |
|  |  | | **Genes in comparison female ctrl vs male ctrl** | | **Differentially expressed genes in comparison male SZ vs male ctrl** | |  | |
| **26 NPC** | **Differentially expressed in female vs male** | | 114 | | 12 | | **Pearson’s Chi-squared test:** | |
|  | **Not differentially expressed in female vs male** | | 36 689 | | 70 | | **p-value (computed from asymptotic chi-squared distribution of the test statistic with Yate’s continuity correction) = 2.708382e-100** | |

| **Supplementary Table 10.** DEGs from SZ: Sex interaction test | | |
| --- | --- | --- |
| Comparison with SZ:Sex interaction term |  | Sex-specific DEGs |
| All samples | HT vs Controls | 538 |
| All samples | ST vs Controls | 99 |
|  |  |  |
| Hoffman et al data: |  |  |
| All hiPSC-NPC samples | COS vs Controls | 78 |
| All hiPSC-neuron samples | COS vs Controls | 145 |

**Supplementary Table 11.** RNA-SEQ Read Alignment.

The alignment statistics are presented below. The overall alignment percentage in all samples was very high, with over 90% of uniquely mapped reads in all samples.

Alignment statistics of the samples showing total number of reads in samples, average length of mapped reads and percentages of reads mapping to unique positions, reads mapping to multiple positions and reads that could not be mapped in the reference.

| Sample name | No of reads | Read length | % Uniquely mapped | % Multi-mapped | % Unmapped |
| --- | --- | --- | --- | --- | --- |
| Control_1_SZ11 | 8576679 | 75 | 92.7 | 6.1 | 1.2 |
| Control_2_SZ12 | 8750425 | 75 | 93.2 | 5.3 | 1.5 |
| Control_3_SZ13 | 10607670 | 75 | 93 | 5.6 | 1.4 |
| Control_4_SZ14 | 7167151 | 75 | 92.69 | 5.71 | 1.59 |
| Control_5_SZ17 | 6106774 | 75 | 92.6 | 6.1 | 1.3 |
| Control_6_SZ18 | 10607461 | 75 | 93 | 5.6 | 1.3 |
| Pair 1_SZ1 | 7709281 | 75 | 92.45 | 5.77 | 1.79 |
| Pair 1_SZ2 | 9885608 | 75 | 91.4 | 5.8 | 2.8 |
| Pair 2_SZ3 | 48967837 | 75 | 91.9 | 5.8 | 2.4 |
| Pair 2_SZ4 | 11768415 | 75 | 92.4 | 6 | 1.7 |
| Pair 3_SZ6 | 5661981 | 75 | 90.47 | 6.57 | 2.97 |
| Pair 3_SZ5 | 10318925 | 75 | 92 | 6.1 | 1.8 |
| Pair 4_SZ8 | 8426905 | 75 | 90.6 | 5.8 | 3.6 |
| Pair 4_SZ7 | 5910513 | 75 | 91.03 | 5.99 | 2.98 |
| Pair 5_SZ9 | 9627376 | 75 | 92.2 | 5.9 | 1.8 |
| Pair 5_SZ10 | 8353359 | 75 | 92.9 | 5.7 | 1.4 |
| Pair 6_SZ16 | 7774231 | 75 | 92.16 | 6.47 | 1.37 |
| Pair 6_SZ15 | 9240384 | 75 | 91.7 | 7 | 1.2 |

**Supplementary Figure 1.** **Characterization of control hiPSC lines**. (A) Representative immunofluorescence images of OCT4, NANOG, TRA 1-81 and SSEA4 from iPS cells grown on top of Matrigel. Scale bars 100 μm. (B) Beta-actin normalized gene expression levels of pluripotency-promoting genes including *NANOG, LIN28, OCT4,* and *SOX2* compared to HS306 embryonic stem cell line used as a positive control. Data are presented as mean ± SEM. (C) Representative karyograms of studied control iPS lines. Female lines: SZ12, SZ13, SZ14 and SZ18. Males lines: SZ11 and SZ17.


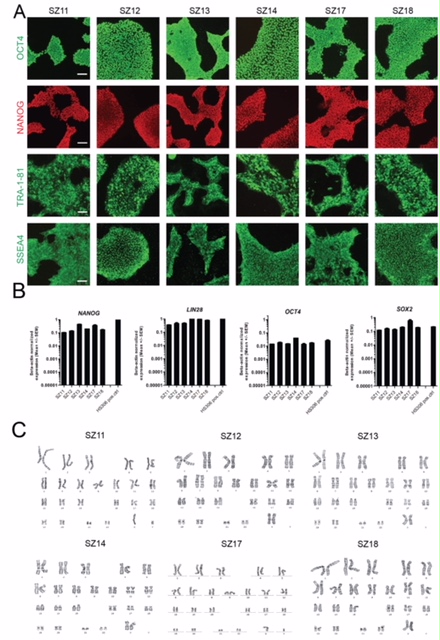


**Supplementary Figure 2. Characterization of female monozygotic twin hiPSC lines**. (A) Representative immunofuoresecence images of OCT4, NANOG, TRA 1-81 and SSEA4 from iPS cells grown on top of Matrigel. Scale bars 100 μm. (B) Representative karyograms of studied iPS lines. Twin pair 1 (SZ1 and SZ2), pair 4 (SZ7 and SZ8) and pair 5 (SZ9 and SZ10). (C) Beta-actin normalized gene expression levels of pluripotency-promoting genes including *NANOG, LIN28, OCT4,* and *SOX2* compared to HS306 embryonic stem cell line used as a positive control. Data are presented as mean ± SEM.


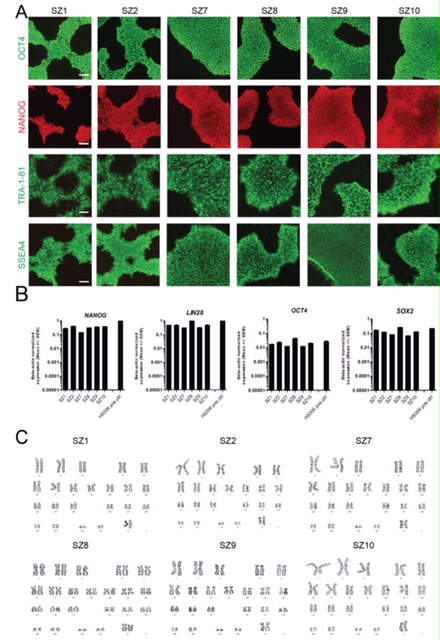


**Supplementary Figure 3. Immunocytochemical analysis of the embryoid body (EBs) formation in female monozygotic twins and healthy female controls.** Representative bright-field images of EBs (scale bar 200 μm) and immunofluorescence images (scale bars 50 µm) of the three embryonic germ layers, including smooth muscle actin (SMA, mesoderm; red), beta III tubulin (TUB3, ectoderm; red) and alpha-fetoprotein (AFP, endoderm; red) from (A) female monozygotic twins and (B) healthy female controls. Nuclei are stained with Hoechst (blue). Female twin pair 1 (SZ1 and SZ2), pair 4 (SZ7 and SZ8) and pair 5 (SZ9 and SZ10). Female healthy control lines: SZ12, SZ13, SZ14 and SZ18.


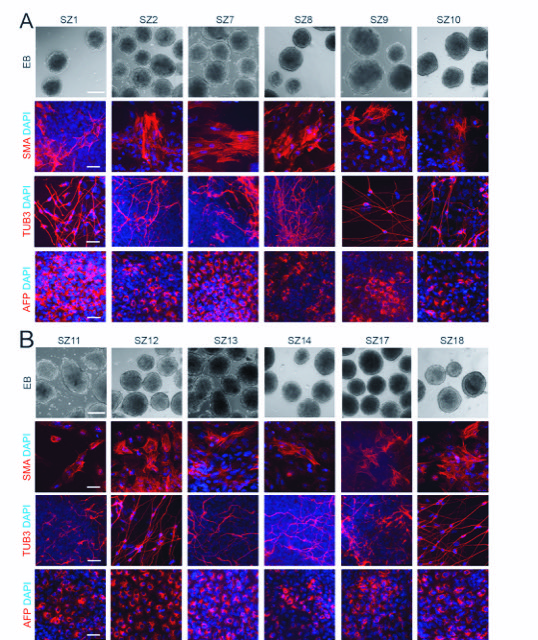


**Supplementary Figure 4. Characterization of male monozygotic twin hiPSC lines.** (A) Representative immunofluorescence images of OCT4, NANOG, TRA 1-81 and SSEA4 from iPS cells grown on top of Matrigel. Scale bars 100 μm. (B) Beta-actin normalized gene expression levels of pluripotency-promoting genes including *NANOG, LIN28, OCT4,* and *SOX2* compared to HS306 embryonic stem cell line used as a positive control. Data are presented as mean ± SEM. (C) Representative bright-field images of EBs (scale bar 200 μm) and immunofluorescence images of the three embryonic germ layers, including smooth muscle actin (SMA, mesoderm; red), beta III tubulin (TUB3, ectoderm; red) and alpha-fetoprotein (AFP, endoderm; red). Nuclei are stained with Hoechst (blue). Scale bars 50 µm. (D) Representative karyograms of studied iPS lines. Twin pair 3 (SZ5 and SZ6) and pair 6 (SZ15 and SZ16).


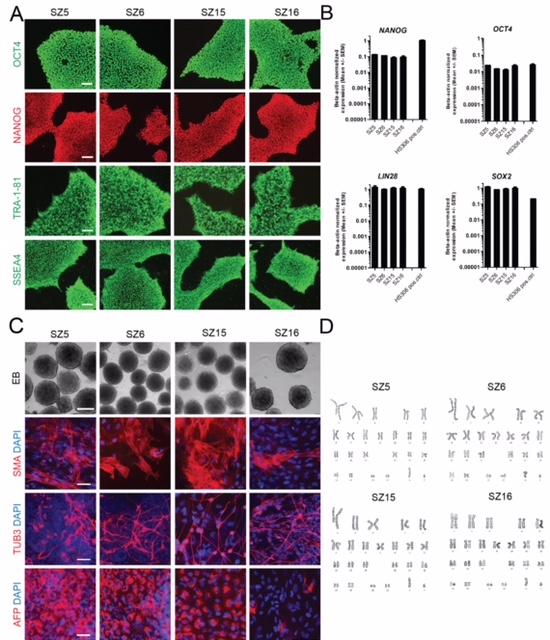


**Supplementary Figure 5. Overlap of sex-specific genes (comparison of healthy males vs. females) in three different datasets.**

**
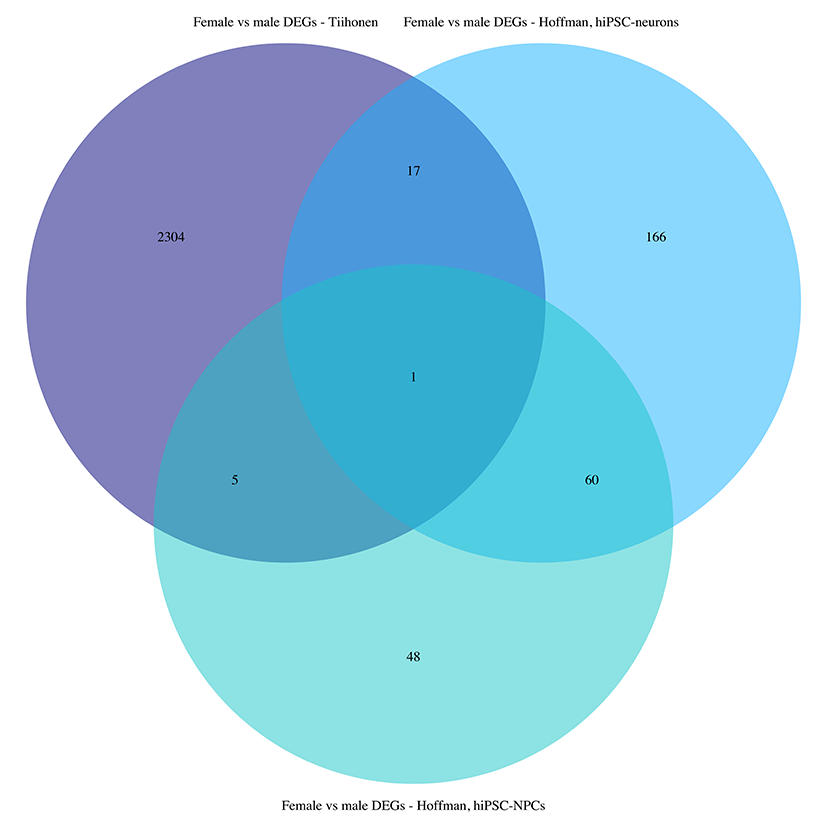
**
