# Supplementary material for: Sex-specific transcriptional and proteomic signatures in schizophrenia
Source: Nat Commun. 2019 Sep 2;10:3933. doi: 10.1038/s41467-019-11797-3 (PMC6718673; doi:10.1038/s41467-019-11797-3)
Supplement: Supplementary file 3 — Reporting Summary [file 41467_2019_11797_MOESM3_ESM.pdf]

nature research

Corresponding author(s): Tiihonen, Lehtonen, Koistinaho

Last updated by author(s): Jul 22, 2019

Reporting Summary

Nature Research wishes to improve the reproducibility of the work that we publish. This form provides structure for consistency and transparency in reporting. For further information on Nature Research policies, see Authors & Referees and the Editorial Policy Checklist .

Statistics

For all statistical analyses, confirm that the following items are present in the figure legend, table legend, main text, or Methods section.

n/a	Confirmed	
☐	☒	The exact sample size (*n*) for each experimental group/condition, given as a discrete number and unit of measurement
☐	☒	A statement on whether measurements were taken from distinct samples or whether the same sample was measured repeatedly
☐	☒	The statistical test(s) used AND whether they are one- or two-sided<br>*Only common tests should be described solely by name; describe more complex techniques in the Methods section.*
☐	☒	A description of all covariates tested
☐	☒	A description of any assumptions or corrections, such as tests of normality and adjustment for multiple comparisons
☐	☒	A full description of the statistical parameters including central tendency (e.g. means) or other basic estimates (e.g. regression coefficient) AND variation (e.g. standard deviation) or associated estimates of uncertainty (e.g. confidence intervals)
☐	☒	For null hypothesis testing, the test statistic (e.g. *F*, *t*, *r*) with confidence intervals, effect sizes, degrees of freedom and *P* value noted<br>*Give P values as exact values whenever suitable.*
☒	☐	For Bayesian analysis, information on the choice of priors and Markov chain Monte Carlo settings
☐	☒	For hierarchical and complex designs, identification of the appropriate level for tests and full reporting of outcomes
☐	☒	Estimates of effect sizes (e.g. Cohen's *d*, Pearson's *r*), indicating how they were calculated

Our web collection on statistics for biologists contains articles on many of the points above.

Software and code

Policy information about availability of computer code

Data collection	*Provide a description of all commercial, open source and custom code used to collect the data in this study, specifying the version used OR state that no software was used.*
Data analysis	GraphPad Prism Software, R package DESeq2, limma R package

For manuscripts utilizing custom algorithms or software that are central to the research but not yet described in published literature, software must be made available to editors/reviewers. We strongly encourage code deposition in a community repository (e.g. GitHub). See the Nature Research guidelines for submitting code & software for further information.

Data

Policy information about availability of data

All manuscripts must include a data availability statement. This statement should provide the following information, where applicable:

- Accession codes, unique identifiers, or web links for publicly available datasets
- A list of figures that have associated raw data
- A description of any restrictions on data availability

Supplementary Tables show raw RNA seq and proteomic data. All the calcium imaging analysis codes were written in Matlab and are available at https://github.com/jussitohka/CalciumImaging. Data available on request from the authors; the RNA seq and proteomic data will also be made available in a public repository (Synapse) after acceptance for publication.

Field-specific reporting

Please select the one below that is the best fit for your research. If you are not sure, read the appropriate sections before making your selection.

- [x] Life sciences
- [ ] Behavioural & social sciences
- [ ] Ecological, evolutionary & environmental sciences

For a reference copy of the document with all sections, see nature.com/documents/nr-reporting-summary-flat.pdf

Life sciences study design

All studies must disclose on these points even when the disclosure is negative.

Sample size	Sample size was 16 independent lines derived from 16 individuals - 6 healthy subjects and 5 twin pairs discordant for schizophrenia.
Data exclusions	Technical failed experiments and outliers were removed before the computation of the summary results.
Replication	Most of the experiments were repeated two to three times.
Randomization	Experimental groups are based on genotype - healthy subject, schizophrenic twin and healthy twin and gender - male and female.
Blinding	Analyses of omics were done in blinded matter.<br>Calcium imaging both the measurements and analyses were done blindly.

Reporting for specific materials, systems and methods

We require information from authors about some types of materials, experimental systems and methods used in many studies. Here, indicate whether each material, system or method listed is relevant to your study. If you are not sure if a list item applies to your research, read the appropriate section before selecting a response.

Materials & experimental systems

n/a	Involved in the study	
[ ]	[x]	Antibodies
[x]	[ ]	Eukaryotic cell lines
[x]	[ ]	Palaeontology
[x]	[ ]	Animals and other organisms
[ ]	[x]	Human research participants
[x]	[ ]	Clinical data

Methods

n/a	Involved in the study	
[x]	[ ]	ChIP-seq
[x]	[ ]	Flow cytometry
[x]	[ ]	MRI-based neuroimaging

Antibodies

Antibodies used

Anti-Oct-4 antibody, clone 10H11.2 (Chemicon; MAB4401)
Human Nanog antibody (R&D Systems; AF1997)
Anti-TRA 1-81, clone TRA-1-81 (Chemicon; MAB4381)
Anti-SSEA4 antibody, clone MC-813-70 (Chemicon; MAB4304)
Monoclonal AFP antibody, clone C3 (Sigma; A8452)
Anti-actin, alpha-SMA antibody (Sigma A5228)
Anti-tubulin ß 3 (Covance; MMS-435P)
Anti-MAP2 antibody, clone AP20 (Chemicon; MAB3418)
Anti-VGLUT1 antibody (Sigma-Aldrich; V0389)
Anti-GABA antibody (Sigma-Aldrich; 2052)

Validation

All antibodies used in this study were validated by company.

Human research participants

Policy information about studies involving human research participants

Population characteristics	Supplementary table 1 summarizes all patients, PANSS score and the patient`s medication.
Recruitment	Patients and their siblings were recruited from the National Twin Register of Finland.
Ethics oversight	Ethics Committee of the Helsinki University Hospital District, licence no. 262/EO/06

Note that full information on the approval of the study protocol must also be provided in the manuscript.
