# Supplementary material for: Sex-specific transcriptional and proteomic signatures in schizophrenia
Source: Nat Commun. 2019 Sep 2;10:3933. doi: 10.1038/s41467-019-11797-3 (PMC6718673; doi:10.1038/s41467-019-11797-3)
Supplement: Supplementary file 4 — Description of Additional Supplementary Files [file 41467_2019_11797_MOESM4_ESM.docx]

**Description of Additional Supplementary Files**

File Name: Supplementary Data 1

Description: DEGs_HT vs Controls

File Name: Supplementary Data 2

Description: DEGs_Female HT vs Female Controls

File Name: Supplementary Data 3

Description: DEGs_Male HT vs Male Controls

File Name: Supplementary Data 4

Description: DEGs_ST vs Controls

File Name: Supplementary Data 5

Description: DEGs_Female ST vs Female Controls

File Name: Supplementary Data 6

Description: DEGs_Male ST vs Male Controls

File Name: Supplementary Data 7

Description: DEGs_ALL ST vs HT

File Name: Supplementary Data 8

Description: DEGs_Female ST vs Female HT

File Name: Supplementary Data 9

Description: DEGs_Male ST vs Male HT

File Name: Supplementary Data 10

Description: DEGs_Controls Female vs Controls Male

File Name: Supplementary Data 11

Description: GOBP and KEGG_ALL ST vs HT

gseaGOBP_comp6; gseaKEGG_comp6

File Name: Supplementary Data 12

Description: GOBP_Female ST vs HT

File Name: Supplementary Data 13

Description: GOBP and KEGG_Male ST vs Male HT

gseaGOBP_comp15; gseaKEGG_comp15

File Name: Supplementary Data 14

Description: GOBP and KEGG_controls Female vs controls Male

orGOBP_comp16; orKEGG_comp16

File Name: Supplementary Data 15

Description: Hoffman_DEG_All Patients vs Controls with sex included in the model_neurons

File Name: Supplementary Data 16

Description: Hoffman_DEG_Female Patients vs Female controls_neurons

File Name: Supplementary Data 17

Description: Hoffman_DEG_Male Patients vs Male controls_neurons

File Name: Supplementary Data 18

Description: Hoffman_DEG_Female controls vs Male controls_neurons

File Name: Supplementary Data 19

Description: Hoffman_DEG_Female controls vs Male controls_NPCs

File Name: Supplementary Data 20

Description: Hoffman_DEG_All Patients vs controls with sex included in the model_NPCs

File Name: Supplementary Data 21

Description: Hoffman_DEG_Female Patients vs Female controls_NPCs

File Name: Supplementary Data 22

Description: Hoffman_DEG_Male Patients vs Male controls_NPCs

File Name: Supplementary Data 23

Description: DEGs_Healthy Twins vs Ctrl_withinteractonTerm_SIGN

File Name: Supplementary Data 24

Description: DEGs_Affected Twins vs Ctrl_withinteractonTerm_SIGN

File Name: Supplementary Data 25

Description: Hoffman_DEGs_All Patients vs Controls_withinteracton_neurons_SIGN

File Name: Supplementary Data 26

Description: Hoffman_DEGs_All Patients vs Controls_withinteracton_NPCs_SIGN

File Name: Supplementary Data 27

Description: DEPs_ALL ST vs HT

File Name: Supplementary Data 28

Description: GO and KEGG_ALL ST vs HT

gseaGO; gseaKEGG

File Name: Supplementary Data 29

Description: DEPs Female ST vs Female HT

File Name: Supplementary Data 30

Description: DEPs Male ST vs Male HT

File Name: Supplementary Data 31

Description: DEP and DEG Female ST vs Female HT

File Name: Supplementary Data 32

Description: DEP_Female Controls vs Male Controls
