# Supplementary material for: Sex-specific transcriptional and proteomic signatures in schizophrenia
Source: Nat Commun. 2019 Sep 2;10:3933. doi: 10.1038/s41467-019-11797-3 (PMC6718673; doi:10.1038/s41467-019-11797-3)
Supplement: Supplementary file 2 — Peer Review [file 41467_2019_11797_MOESM2_ESM.pdf]

Reviewers' comments:

Reviewer #1 (Remarks to the Author):

The authors use iPS-derived neurons from twins discordance for Schizophrenia (Sz) to identify gene expression and proteomic signatures of disease. The authors nicely demonstrate that using twin pairs can remove some of the noise due to genetic heterogeneity.

The authors' findings of sex specific case-control difference is intriguing, but they only demonstrate this in a single dataset. The authors needs to show that their findings are robust and reproducible across multiple schizophrenia datasets. I suggest Hoffman, et al, 2017, for iPS and Fromer, et al., Gandal, et al, Science 2018, Jaffe, et al, Nat Neuro, 2018 for post mortem human brain.

Major Comments

1. Statistical analysis of gene expression data
I am concerned that the findings of sex-specific effects on Schizophrenia may be driven by small sample size. The accuracy of p-values can be a serious issues at small sample size (Law, et al. Genome Biology 2014). In fact, in this study the number of differential expressed genes is related to the size of the analysis: the within-sex analyses give more differential expressed genes, that the combined analysis. Performing the analysis in males the females, and then intersecting the results can be very statistically unstable and can give many false positives.

In order to robustly identify sex-specific genes, the analysis should be performed using a combined model and then test the interaction term: ~ Sz + Sex + Sz:Sex. A significant interaction term, Sz:Sex, indicates the that Sz effect sizes differ between males and females. It does not appear that this test was performed.

How was the analysis of the twin controls performed? Was a paired analysis used (comparing each sample to its twin) using the formula: ~ Sz + Family. Or was an unpaired analysis done comparing only the group means of each group: ~ Sz. I strongly recommend using a paired analysis.

Comparing male controls to female controls identified 2327 differentially expressed genes. That is a huge number of genes. Is that plausible given the findings from sex differences observed in larger studies? Is this findings reproduced in a large dataset such as CommonMind, or this this attributable to the small sample size?

Figure 1E,F What is normalized enrichment score? Is that more significant than expected under the null?

2. Need to replicate findings in other schizophrenia datasets
It is not clear how the findings from this analysis relate to the findings from other, much larger datasets. I would strongly recommend comparing the disease and sex differential expression signatures to the findings of Fromer, et al, Nat Neuro, 2016, Jaffe, et al, Nat Neuro, 2018, Hoffman et al, Nat Comm, 2017; Gandal, et al. Science, 2018. These references have released RNA-seq counts or differential expression signatures to facilitate this comparison.

3. Description of methods is not adequate.
In the main manuscript the methods section is only a single paragraph. There is more information in the supplement, but it is still short of details. For the RNA-seq analysis the authors should describe what software was used to perform read alignment and gene quantification. What reference genome and what gene models were used? Single or paired end? How many reads were obtained and how man mapped to the genome. Did PCA or hierarchal clustering identify any outliers? Were these samples

processed in batches and if so how was that ache effect accounted for in the statistical analysis?

For the proteomics analysis, the log2 fold change cutoff of >0.379 seems very arbitrary and is not explained

4. Batch effects in proteomics analysis.
Since SZ risk is polygenic, we expect expression and protein signatures of disease to be subtle. This is supported by previous transcriptome studies (Fromer, et al, Nat Neuro, 2016, Jaffe, et al, Nat Neuro, 2018, Hoffman et al, Nat Comm, 2017; Gandal, et al. Science, 2018). The complete separation of case, twin controls and healthy controls is concerning and suggests a very strong batch effect that must be accounted for in the differential analysis.

Minor Comments
Do the authors have a data release plan for the non-genetic data? Gene Expression Omnibus or Synapse are good options.

Reviewer #2 (Remarks to the Author):

This manuscript reports the surprising observation that sex-specific signatures of Schizophrenia (SCZ) can be seen in discordant twin pair analysis of gene expression and proteomics in only 5 pairs of twins. A sixth pair was excluded from analysis due to phenotypic blurring between effected and uneffected. The observation is intriguing, and could have profound impact on the treatment of SCZ if indeed the gene expression differences underlying disease are disease-specific. A few concerns were raised by review though, that need to be addressed to better understand the rigor and value of these observations.

• Authors in many instances seem to equate the expression phenotypes in iPSC derived neurons from the subjects with the actual subject - e.g. Pg 4 "... we compared affected twins with their healthy co-twins, and found decreased expression of LHX1...". Indeed, the claim that the ST vs HT comparison enables distinguishing "actual clinical illness" from "increased family risk" is unsubstantiated, nor even theoretically justified. Rather this comparison may distinguish individual risk vs family risk (e.g. distinguishing individual genetic/epigenetic differences between the ST and HT).

• Information about batch processing is lacking in the methods. Namely, were all 6 twin pairs and 6 controls differentiated together and RNA and protein harvested from this same batch - or were these done in multiple cohorts. If not done all together, how were the cohorts organized. This information is pertinent as cohort effects may influence expression differences as the shared media within an experimental cohort can influence neuronal phenotype.

• It is unclear if the gene expression validation qPCR is from the same RNA or differentiation as the RNA used for the RNAseq experiment. This validation should be done with an independent differentiation; and even more rigorous should be perform from an independent iPSC line from the same subject. Otherwise, it is unclear if the apparent twin differences are due to real genetics/epigenetic differences between effected/uneffected vs iPSC line specific effects.

•. It is unclear the statistical/analytical definition of a "sex-specific DEGs", e.g. is there a statistical cut off for saying what is "sex-specific", and what minimal difference in the other sex disqualifies a DEG from being labeled as "sex-specific". The supplemental methods or main paper are not clear on this critical point.

•. The proteomic validation of differences between HT and ST samples is a strength of this manuscript - indeed the PCA separation by disease status and the observation that sex-segregation improved the number of SCZ associated DEGs is intriguing and a valuable observation to report. However, the strength (or not) of the sex effect would be further reveal if the sex (subject) of the individual HT and ST lines were labeled in Fig 3D.

• The authors state that (pg 6 - "iPSC-derived neurons at this phase correspond to the developmental stage of the early second trimester of pregnancy") yet, they also state that sex-specific gene expression become pronounced as teenage/early 20s ages. Further, the authors conclude that differences in calcium responses that respond (or not) to clozapine are related to SCZ and TRS; yet immature neurons are not the stage at which SCZ occurs, thus greater caution of interpreting these data are necessary. Also TRS vs non-TRZ SCZ are also male vs female in the authors data. This distinction, why clear in some aspects of the paper, need to be duly considered in the context of Figure 4 as well. This fact that sex and TRS perfectly overlap makes it impossible to distinguish the role of sex vs TRS in the effects reported - e.g. even the female HT do not account for this as the risk associated with both sex or TRS could have instead been associated with familial risk shared between the twins; thus it could still be that either sex or TRS risk or both explain the observations. More rigorous consideration of this is necessary. Confirmation of the gene/pathway specific effects in both male and female TRS, vs more male and female SCZ lines seems critical to determine the role of both.

• Figure 4E-F is difficult to interpret and undersrtand what the different colors points with variance, vs the boxes mean and what samples the correspond to - legend does not explain well enough. This figure needs to be improved for clarity.

• CNV in fibroblasts; should have been confirmed in the iPSC parental line from the observed. But more improtantly, the authors seem to completely ignore the power of the sib-pairing the in the CNV analysis - simply lumping all affected and unaffected together, vs incorporating pairedness into the model. As this is argued to be the basis for the improved power, why not apply this to the CNV data as well? Also, by not doing this (at least as explained) there seems to not be a mapping of the gene expression differences belonging to specific subjects lines to the gene expression changes in the individual and paired group.

Nature Communications
RE: Manuscript NCOMMS-19-08565, "Sex-specific transcriptional and proteomic signatures in schizophrenia"

Dear Editor,

We thank you and the reviewers for the constructive criticism on our manuscript "Sex-specific transcriptional and proteomic signatures in schizophrenia". We have addressed all the issues raised by the reviewers, and modified our manuscript as follows:

REVIEWER #1

The authors use iPS-derived neurons from twins discordance for Schizophrenia (Sz) to identify gene expression and proteomic signatures of disease. The authors nicely demonstrate that using twin pairs can remove some of the noise due to genetic heterogeneity.

The authors' findings of sex specific case-control difference is intriguing, but they only demonstrate this in a single dataset. The authors needs to show that their findings are robust and reproducible across multiple schizophrenia datasets. I suggest Hoffman, et al, 2017, for iPS and Fromer, et al., Gandal, et al, Science 2018, Jaffe, et al, Nat Neuro, 2018 for post mortem human brain.

Major Comments

1. Statistical analysis of gene expression data
I am concerned that the findings of sex-specific effects on Schizophrenia may be driven by small sample size. The accuracy of p-values can be a serious issues at small sample size (Law, et al. Genome Biology 2014). In fact, in this study the number of differential expressed genes is related to the size of the analysis: the within-sex analyses give more differential expressed genes, that the combined analysis. Performing the analysis in males the females, and then intersecting the results can be very statistically unstable and can give many false positives.

In order to robustly identify sex-specific genes, the analysis should be performed using a combined model and then test the interaction term: ~ Sz + Sex + Sz:Sex. A significant interaction term, Sz:Sex, indicates the that Sz effect sizes differ between males and females. It does not appear that this test was performed.

OUR RESPONSE: We thank for this valuable comment. We have now studied SZ effects by testing the interaction term ~ SZ + Sex + SZ:Sex, as suggested. In both ST vs control and HT vs control comparisons, the results with respect to SZ:Sex interaction were significant with 99 and 538 DEGs, respectively, indicating sex-specific differences in the illness and familial risk effect. The same interaction analysis was also done using

Hofmann et al. data, resulting in 78 DEGs that indicates sex-specific difference in the COS vs control effect. We have now also analyzed gene expression by including sex in the model, and that resulted in larger number of DEGs in the comparison between unaffected twins vs. controls, and between affected twins vs. controls (see Supplementary Table 22; new Supplementary Tables 8 and 11). Both our dataset and dataset by Hoffman et al. show that the number of DEGs is larger in the comparison of male patients vs. male controls and female patients vs. female controls than when all patients are compared with all controls, despite of lower number of subjects when including males only or females only. This may be explained by the fact that DEGs in male and female comparisons are not the same (the effect can be even opposite for specific genes), the results are diluted or counterbalanced when males and females are analyzed together, and the number of DEGs gets smaller despite higher number of subjects. (See Results, Reproducibility paragraph; modified Figure 1G, and Figure 2E, new Supplementary Table 8, new Supplementary Table 11).

How was the analysis of the twin controls performed? Was a paired analysis used (comparing each sample to its twin) using the formula: ~ Sz + Family. Or was an unpaired analysis done comparing only the group means of each group: ~ Sz. I strongly recommend using a paired analysis.

OUR RESPONSE: We have now stated more clearly that we have used paired analysis (Supplementary Methods, Gene Expression Analysis).

Comparing male controls to female controls identified 2327 differentially expressed genes. That is a huge number of genes. Is that plausible given the findings from sex differences observed in larger studies? Is this findings reproduced in a large dataset such as CommonMind, or this this attributable to the small sample size?

OUR RESPONSE: Microarray analysis by Trabzuni el al. (2013) showed widespread sex differences in gene expression in adult human brain, being detectable in all major brain regions and involving 2.5% of all expressed genes. Mayne et al. (2016) have studied sex differences in gene expression in various tissues using RNA-seq and found the greatest sex differences in gene expression in the brain, specifically in the anterior cingulate cortex (1818 DEGs). hiPSC-neurons resemble the gene expression of fetal forebrain tissue (Hoffman et al. 2017), and O'Brien et al. (2018) showed that there were sex differences in the expression of 2558 autosomal genes and 155 X-chromosomal genes at FDR < 0.1. Because gene expression fluctuates markedly during development and the lifespan, the comparison with other datasets should be done by matching the developmental phase of the individuals. Since CommonMind data are from postmortem adult brains, the gene expression is not comparable to developing fetal neurons. Therefore, we compared the gene expression in the dataset by Hoffman et al. Nat Commun 2017 (the only available iPSC neuron dataset). Results, Reproducibility paragraph.

Venn-diagram in Supplementary Figure 5 shows the overlap of sex-specific genes in neurons in our dataset and in neurons and NPCs in the Hoffman et al. dataset. The number of sex-specific genes was smaller (244) in hiPSC-derived neurons in the Hoffman et al. dataset (with NPC-derived neurons differentiated for 6 weeks) than in our hiPSC-derived neurons (NPC-derived neurons differentiated for 10 weeks), with 18 genes overlapping with our sex-specific genes. The corresponding numbers were 114 and 6 concerning hiPSC-derived NPCs. This suggests that the number of sex-specific genes increases as function of time during maturation of cells.

Figure 1E,F What is normalized enrichment score? Is that more significant than expected under the null?

OUR RESPONSE: The GSEA algorithm calculates an enrichment score (ES) that reflects the degree to which a gene is over-represented at the top or bottom of a ranked list of genes. The algorithm walks down the ranked gene list and increases a running-sum statistic when a gene is in the gene set and decreases the statistic when it is not. Thus, the ES is the maximum deviation from zero encountered in walking the list, with positive ES indicating gene set enrichment at the top of the list and a negative ES indicating gene set enrichment at the bottom of the list. The normalised enrichment score (NES) accounts for differences in gene set size and in correlations between gene sets and the expression dataset and can be used to compare analysis results across gene sets. (Source: https://software.broadinstitute.org/gsea/doc/GSEAUserGuideTEXT.htm)

We have added a short description of the NES in the Supplementary Methods.

2. Need to replicate findings in other schizophrenia datasets
It is not clear how the findings from this analysis relate to the findings from other, much larger datasets. I would strongly recommend comparing the disease and sex differential expression signatures to the findings of Fromer, et al, Nat Neuro, 2016, Jaffe, et al, Nat Neuro, 2018, Hoffman et al, Nat Comm, 2017; Gandal, et al. Science, 2018. These references have released RNA-seq counts or differential expression signatures to facilitate this comparison.

OUR RESPONSE: We thank the reviewer for this important and valuable comment. We totally agree that replication is the real thing. We have now replicated our findings by using data from Hoffman et al. 2017 which is the only iPSC study including patients with schizophrenia (other studies include only postmortem samples from patients with schizophrenia). These results are now shown in Results, Reproducibility paragraph, and show that our findings on expression of sex-specific genes are robust and replicable.

3. Description of methods is not adequate.
In the main manuscript the methods section is only a single paragraph. There is more information in the supplement, but it is still short of details. For the RNA-seq analysis the authors should describe what software was used to perform read alignment and gene quantification. What reference genome and what gene models were used? Single or paired end?

OUR RESPONSE: Single-end RNA-seq reads were aligned to human GRCh38 (hg38) genome assembly with Ensembl GRCh38.90 transcript annotations using STAR aligner, version 2.5.2 [Dobin et al., 2013]. Gene-level read counts were obtained simultaneously with the alignment process. For visual exploration of the data, the obtained read counts were normalized using regularized log transformation function of DESeq2 R package [Love et al., 2014, v 1.14.1], which transforms the count data to the log2 scale in a way that minimizes differences between samples for rows with small counts and also normalizes the data with respect to library size. Samples were visually inspected using principal component analysis (PCA) and a Pearson's correlation heatmap before proceeding to differential expression analysis.

CHANGES IN THE MANUSCRIPT: More detailed RNA-seq analysis methods are described in the Methods supplement.

How many reads were obtained and how man mapped to the genome.

OUR RESPONSE: Quality of the RNA-seq reads was inspected using FastQC software [Andrews, 2010]. General statistics table of the FastQC analysis results from all samples were generated using R package fastqcr [Kassambara, 2017]. The quality of the reads was very good, but some reads contained over-represented sequences that appeared to be adapter sequences. These were removed by trimming using cutadapt tool [Martin, 2011] with minimum length of processed reads set to 35. FastQC analysis was performed again for the trimmed reads.

CHANGES IN THE MANUSCRIPT: Alignment statistics table (shown below) is provided as a Supplementary Table 43.

Supplementary Table 43. RNA-SEQ Read Alignment.

The alignment statistics are presented below. The overall alignment percentage in all samples was very high, with over 90% of uniquely mapped reads in all samples.

Alignment statistics of the samples showing total number of reads in samples, average length of mapped reads and percentages of reads mapping to unique positions, reads mapping to multiple positions and reads that could not be mapped in the reference.

Sample name	No of reads	Read length	% Uniquely mapped	% Multi-mapped	% Unmapped
Control_1_SZ11	8576679	75	92.7	6.1	1.2

Control_2_SZ12	8750425	75	93.2	5.3	1.5
Control_3_SZ13	10607670	75	93	5.6	1.4
Control_4_SZ14	7167151	75	92.69	5.71	1.59
Control_5_SZ17	6106774	75	92.6	6.1	1.3
Control_6_SZ18	10607461	75	93	5.6	1.3
Pair 1_SZ1	7709281	75	92.45	5.77	1.79
Pair 1_SZ2	9885608	75	91.4	5.8	2.8
Pair 2_SZ3	48967837	75	91.9	5.8	2.4
Pair 2_SZ4	11768415	75	92.4	6	1.7
Pair 3_SZ6	5661981	75	90.47	6.57	2.97
Pair 3_SZ5	10318925	75	92	6.1	1.8
Pair 4_SZ8	8426905	75	90.6	5.8	3.6
Pair 4_SZ7	5910513	75	91.03	5.99	2.98
Pair 5_SZ9	9627376	75	92.2	5.9	1.8
Pair 5_SZ10	8353359	75	92.9	5.7	1.4
Pair 6_SZ16	7774231	75	92.16	6.47	1.37
Pair 6_SZ15	9240384	75	91.7	7	1.2

Did PCA or hierarchal clustering identify any outliers?

OUR RESPONSE: Samples were visually inspected using principal component analysis (PCA) and a Pearson's correlation heatmap before proceeding to differential expression analysis.

These analyses were both performed to data as a final method to ensure data quality. In the principal component analysis using all data, the two principal components explained 53.8 % of the variance between samples. According to both the visualisation of the principal component analysis and Pearson's correlation analysis, no clear clustering based on any of the defined variables was observed, with the exception of male control samples and female control samples, which clustered by sex. The PCA result suggests that the differences found between sample groups will be small with the exception of differences between male and female control samples. No clear outlier samples were observed in PCA.

Were these samples processed in batches and if so how was that ache effect accounted for in the statistical analysis?

OUR RESPONSE. The RNA-seq samples were processed as a single batch.

For the proteomics analysis, the log2 fold change cutoff of >0.379 seems very arbitrary and is not explained

OUR RESPONSE: In addition to statistical significance at p<0.05, 30% (= log2-fold change) increase or decrease in protein expression was required for regarding protein as differentially expressed.

CHANGES IN THE MANUSCRIPT: Log2 FC cutoff value was replaced by the above explanation in the methods supplement.

4. Batch effects in proteomics analysis.
Since SZ risk is polygenic, we expect expression and protein signatures of disease to be subtle. This is supported by previous transcriptome studies (Fromer, et al, Nat Neuro, 2016, Jaffe, et al, Nat Neuro, 2018, Hoffman et al, Nat Comm, 2017; Gandal, et al. Science, 2018). The complete separation of case, twin controls and healthy controls is concerning and suggests a very strong batch effect that must be accounted for in the differential analysis.

OUR RESPONSE: All iPSC lines included in proteomic analysis were differentiated to neurons, processed for protein analysis, as well as analysed simultaneously. During analysis, samples were distributed into 2 TMT® 10 plexes, having 3 samples of each group into one TMT®plex. Altogether 18 samples were run including 6 twin pairs and 6 controls. Each TMT®plex contained a reference channel created by pooling all 18 samples. The analysis was performed at the peptide, phosphopeptide and protein levels and TMT®plex batch effect was successful removed before final analysis. Thus, there is no batch effect on our data.

CHANGES IN THE MANUSCRIPT: More detailed descriptions of the proteomics PCA plots have been included in the methods supplement as well as in the Figure 3 legend.

Minor Comments
Do the authors have a data release plan for the non-genetic data? Gene Expression Omnibus or Synapse are good options.

OUR RESPONSE: We plan to release the data after the embargo date.

REVIEWER #2:

This manuscript reports the surprising observation that sex-specific signatures of Schizophrenia (SCZ) can be seen in discordant twin pair analysis of gene expression and proteomics in only 5 pairs of twins. A sixth pair was excluded from analysis due to phenotypic blurring between effected and uneffected. The observation is intriguing, and could have profound impact on the treatment of SCZ if indeed the gene expression differences underlying disease are disease-specific. A few concerns were raised by review though, that need to be addressed to better understand the rigor and value of these observations.

• Authors in many instances seem to equate the expression phenotypes in iPSC derived neurons from the subjects with the actual subject - e.g. Pg 4 "... we compared affected twins with their healthy co-twins, and found decreased expression of LHX1...". Indeed, the claim that the ST vs HT comparison enables distinguishing "actual clinical illness" from "increased family risk" is

unsubstantiated, nor even theoretically justified. Rather this comparison may distinguish individual risk vs family risk (e.g. distinguishing individual genetic/epigenetic differences between the ST and HT).

OUR RESPONSE: We have now modified the wording concerning "actual clinical illness" and "increased family risk". (Page 4, Transciptional signatures related to clinical illness paragraph)

• Information about batch processing is lacking in the methods. Namely, were all 6 twin pairs and 6 controls differentiated together and RNA and protein harvested from this same batch - or were these done in multiple cohorts. If not done all together, how were the cohorts organized. This information is pertinent as cohort effects may influence expression differences as the shared media within an experimental cohort can influence neuronal phenotype.

OUR RESPONSE: All 5 twin pairs and 6 healthy control lines were differentiated simultaneously. Also RNA and protein samples were harvested together. Thus, there is no batch effect affecting the data. We have now clarified this method part in Supplementary Material. See also our response to comment 4 by Reviewer #1.

• It is unclear if the gene expression validation qPCR is from the same RNA or differentiation as the RNA used for the RNAseq experiment. This validation should be done with an independent differentiation; and even more rigorous should be perform from an independent iPSC line from the same subject. Otherwise, it is unclear if the apparent twin differences are due to real genetics/epigenetic differences between effected/uneffected vs iPSC line specific effects.

OUR RESPONSE: The gene expression validation qPCR has been performed from both the same and another neuronal differentiation than the RNA used for the RNAseq experiment. Underneath are updated examples with RNA from another neuronal differentiation, including PRKG2, which came up with significantly different mRNA expression between affected twins and healthy controls and between affected and unaffected twins and healthy controls, when we revised the statistical analysis of gene expression data according to the recommendation by reviewer 1. Underneath is also an example of validation of qPCR from hiPSC-derived neurons obtained from the second clone of a female twin pair (SZ1 and SZ2) and healthy control SZ13 hiPSCs.

In the case of substantial line-to-line variation within each subject, inclusion of only one line per subject could in principal result in larger than actual variation within the study groups compared to study material when several lines per each subject are included for verification. In other words, our results based on only one line per subject could in principal mask some gene expression differences that could be detected when line-to-line variation is excluded by using several lines per each subject. Therefore, our study based on one line per each subject does not result in false positive findings, but rather suggests that the observed differences in gene/protein expression could even be more

significant, had the possible line-to-line variation excluded. Our validation example with the second lines described above suggest that line-to-line variation in our iPSC material is not significant.

Example Figure: qPCR validation of several genes from different comparison sets using hiPSC-derived neurons. Repeated qPCR (A) from different neuronal differentiation batch, (B) from different clones derived from twin female pair SZ1, SZ2 and control SZ13. (C) Validation of PRKG2 gene. The data are shown as mean +/- SEM. The expression of genes in female (red circles) and in males (blue circles).

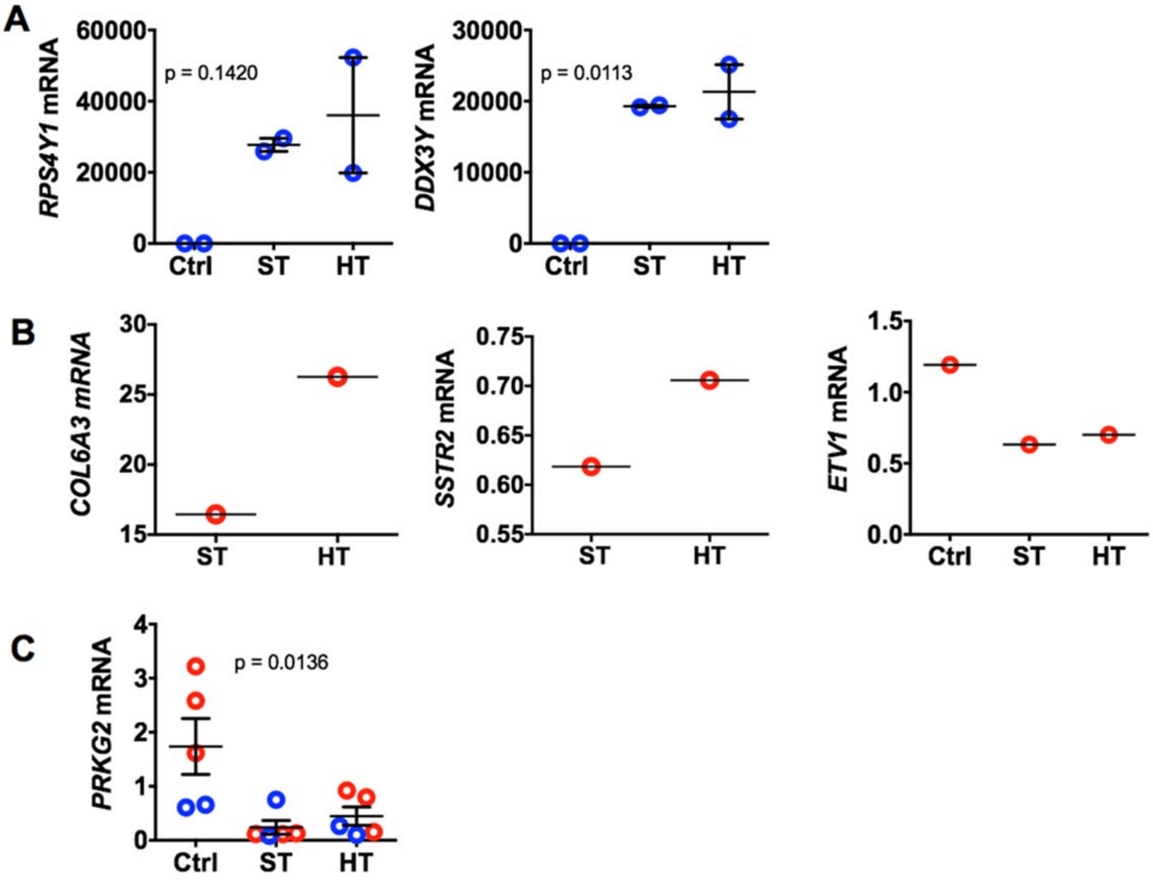

•. *It is unclear the statistical/analytical definition of a "sex-specific DEGs", e.g. is there a statistical cut off for saying what is "sex-specific", and what minimal difference in the other sex disqualifies a DEG from being labeled as "sex-specific". The supplemental methods or main paper are not clear on this critical point.*

OUR RESPONSE: Genes that were differentially expressed between healthy male controls and healthy female controls (with adj. p-value < 0.05 and at least 2-fold expression change) were analysed, and these were referred to as 'sex-specific genes'. DEGs from the

other comparisons were labeled as sex-specific, if they were found to belong to the group of genes expressed differentially in males and females. This type of rough classification was repeated using a larger hiPSC dataset from Hoffman et al, and the results were concordant. Sex-related differences in clinical illness as well as familial risk gene expression signatures were further studied by analysing the statistical significance of the interaction term SZ:sex using both data. These analyses resulted in significant differences in both datasets.

CHANGES IN THE MANUSCRIPT: A sentence explaining the sex-specific genes has been added in the 'Sex-specific gene expression' paragraph in the manuscript.

• The proteomic validation of differences between HT and ST samples is a strength of this manuscript - indeed the PCA separation by disease status and the observation that sex-segregation improved the number of SCZ associated DEGs is intriguing and a valuable observation to report. However, the strength (or not) of the sex effect would be further reveal if the sex (subject) of the individual HT and ST lines were labeled in Fig 3D.

OUR RESPONSE: We have now modified Figure 3D as suggested.

• The authors state that (pg 6 - "iPSC-derived neurons at this phase correspond to the developmental stage of the early second trimester of pregnancy") yet, they also state that sex-specific gene expression become pronounced as teenage/early 20s ages. Further, the authors conclude that differences in calcium responses that respond (or not) to clozapine are related to SCZ and TRS; yet immature neurons are not the stage at which SCZ occurs, thus greater caution of interpreting these data are necessary. Also TRS vs non-TRZ SCZ are also male vs female in the authors data. This distinction, why clear in some aspects of the paper, need to be duly considered in the context of Figure 4 as well. This fact that sex and TRS perfectly overlap makes it impossible to distinguish the role of sex vs TRS in the effects reported - e.g. even the female HT do not account for this as the risk associated with both sex or TRS could have instead been associated with familial risk shared between the twins; thus it could still be that either sex or TRS risk or both explain the observations. More rigorous consideration of this is necessary. Confirmation of the gene/pathway specific effects in both male and female TRS, vs more male and female SCZ lines seems critical to determine the role of both.

OUR RESPONSE: We have now added text on the role of sex vs. TRS in Results, Electrophysiological analyses paragraph, last 4 lines.

• Figure 4E-F is difficult to interpret and undersrtand what the different colors points with variance, vs the boxes mean and what samples the correspond to - legend does not explain well enough. This figure needs to be improved for clarity.

OUR RESPONSE: The comment is well taken. We have now clarified the meaning of the figure 4E-F colours, boxes and other symbols used in the figure legends. We have also added details of the analysis and additional references into the Material and method part.

We have now rephrased the legend as follows:

"Figure 4. Spontaneous neuronal activity and neuronal calcium imaging of affected and unaffected twins. (A) hiPSC-derived neurons stained for VGLUT1 (red), TUB3 (green) and GABA (red). (B) Regular spontaneous neuronal reactivity and (C) membrane current induced by 100 µM GABA or by 100 µM glutamate with 10 µM co-agonist glycine in neuronal cultures of healthy control subjects. Scale bar 20 µm. (D) Representative calcium traces and quantification from neurons of (E) treatment-resistant and (F) non-treatment resistant (patients that respond to standard antipsychotics) schizophrenia before and after clozapine treatment. Glu+Gly (no Mg) indicates NMDA specific responses. ST, affected twin; HT, unaffected twin. In panels E and F, results are presented representing both levels of the hierarchical two-level random effects analysis. The cyan line and black box represent, respectively, the mean and its standard error of the upper, population level to which p-values and Z-statistics refer to. Colored dots and their whiskers refer to the first level analyses of individual subject pairs. Dots represent the average differences in response between unaffected and affected twin. Whiskers represent the standard errors of the averages. Population level mean (cyan line) is the inverse variance weighted mean of the subject-pair-wise average differences. . The subject pairs are in Panel E: Red = Pair 1, Black = Pair 4, Blue = Pair 5, and in Panel F: Red = Pair 3, Blue = Pair 6. *** $p<0.001$."

• CNV in fibroblasts; should have been confirmed in the iPSC parental line from the observed. But more improtantly, the authors seem to completely ignore the power of the sib-pairing the in the CNV analysis - simply lumping all affected and unaffected together, vs incorporating pairedness into the model. As this is argued to be the basis for the improved power, why not apply this to the CNV data as well? Also, by not doing this (at least as explained) there seems to not be a mapping of the gene expression differences belonging to specific subjects lines to the gene expression changes in the individual and paired group.

OUR RESPONSE: We decided to analyze CNVs using the DNA extracted from the fibroblasts in order to compare the actual genomic differences between the subject materials used for RNA-seq analyses. The CNVs have been analyzed pair-wise in twins. The concordant and de novo CNVs are separated by comparing the affected and unaffected twins.

It is important to note that large CNVs containing several genes are extremely rarely different between twin pairs (Xu C et al., Twin Res Hum Genet. 2017; 20:60-65) thus limiting the further potential analyses that could be performed expecting conclusive results.

Moreover, CNVs provide a description of only one potential level of genomic variation. The concordant CNVs were described at a thematic level to provide description of the genomic background (especially paying attention to SMAD pathway), but, naturally, concordant CNVs are same in both twins, preventing thus inter-twin comparison. 107 of all 164 detected CNVs were completely concordant and observed as identical between affected and unaffected twins. Although the de novo variants were followed further, however, with very limited findings were observed:

- **The 26 de novo CNVs detected in affected twins but not in unaffected twins, were located on areas corresponding to 84 genes. None of these 84 genes matched with genes that showed a significantly different expression between affected and unaffected twin (data not shown).**

- **Direct pairing data of CNVs and mRNAs is meaningful with genes along de novo CNVs in affected twins, when observed in at least two twin pairs. CNTRL and TMEM72-AS1 (located in chromosomes 9 and 10 respectively), were in loss de novo CNVs in two affected twins. However, there was no significant difference in the mRNA expression levels of these genes between the affected and unaffected twins. Additionally, PDPR in chromosome 16 was located in a gain CNV in one affected twin and in a loss CNV in another affected twin. No conclusions can be drawn with so few findings. Therefore, the analysis is not included into the manuscript.**

We subjected the 26 de novo CNVs in affected twins to a thematic analysis of the genes to determine the mechanisms by which the CNVs may affect these individuals, paying attention particularly to relevant neurodevelopmental mechanisms. Altogether, there was only one enriched KEGG pathway, “neuroactive ligand-receptor interaction” (p=0.048) corresponding to glutamatergic GRID2 and neuropeptide Y receptor NPY4R (also represented as the CH17-360D5.1 variant). There were no significant mRNA expression differences between pairs in case of GRID2 or NPY4R in the tested model (data not shown), despite the well-established involvement of glutamatergic system in schizophrenia.

Altogether, our material had 5 twin pairs discordant for schizophrenia. Pairing genomic data directly on gene expression for statistically significant mechanistic explanation would require substantially more study subjects. Because the pairwise analysis of mRNA vs CNV analysis would be statistically very weak with so few subjects, we decided not to include it into the manuscript. When interpreting mRNA data, it was observed that the highly altered gene expressions (mRNA) between iPSC lines of affected and unaffected

twins were not the same as those observed in CNV analysis. Therefore, it cannot be concluded that that observed CNVs are a direct cause of gene expression changes with reasonable confidence.

To describe the inter-twin differences in CNV data, we edited the manuscript result section with the following text:

"The 26 de novo CNVs found in affected twins but not in unaffected twins were located on areas corresponding to 84 genes, and the 18 de novo mutations found in unaffected twins but not in affected twins were located in areas corresponded to 213 genes. Among those 26 de novo mutations in affected twins only, there was only one enriched KEGG pathway, "neuroactive ligand-receptor interaction" (p=0.048), corresponding to glutamatergic GRID2 and neuropeptide Y receptor NPY4R, also represented as CH17-360D5.1 variant in the list. When compared to transcriptomic data, there were no significant mRNA expression differences between the pairs in case of GRID2 or NPY4R in the tested model, despite the well-established involvement of glutamatergic system in schizophrenia."

References:

Hoffman GE, Hartley BJ, Flaherty E, Ladran I, Gochman P, Ruderfer DM, Rapoport J, Sklar P, Brennand KJ. 2017. Transcriptional signatures of schizophrenia in hiPSC-derived NPCs and neurons are concordantwith post-mortem adult brains. Nat Commun. 8(1):2225.

Mayne BT, Bianco-Miotto T, Buckberry S, Breen J, Clifton V, Shoubridge C, Roberts CT. 2016. Large Scale Gene Expression Meta-Analysis Reveals Tissue-Specific, Sex-Biased GeneExpression in Humans. Front Genet. 7:183

O'Brien HE, Hannon E, Jeffries AR, Davies W, Hill MJ, Anney RJ, O'Donovan MC, Mill J, Bray NJ. 2018. Sex differences in gene expression in the human fetal brain. bioRxiv. doi: https://doi.org/10.1101/483636.

Trabzuni D, Ramasamy A, Imran S, Walker R, Smith C, Weale ME, Hardy J, Ryten M; North American Brain Expression Consortium. 2013. Widespread sex differences in gene expression and splicing in the adult human brain. Nat Commun. 4:2771.

REVIEWERS' COMMENTS:

Reviewer #2 (Remarks to the Author):

The authors have satisfactorily addressed all prior concerns raised in review. The addition of the 'reproducibility' section and it's analysis further strengthens the rigor of their sex-specific findings. The study has strong potential to be impactful to the field.

Minor comments:
2nd paragraph of 'reproducibility' section has misspelling in first sentence. Should be "healthy". In addition, there seems to be some font changes in portions of the text.

Signed,
Aaron Bowman
Purdue University

REVIEWERS COMMENTS:

Reviewer #2 (Remarks to the Author):

The authors have satisfactorily addressed all prior concerns raised in review. The addition of the 'reproducibility' section and it's analysis further strengthens the rigor of their sex-specific findings. The study has strong potential to be impactful to the field.

Minor comments: 2nd paragraph of 'reproducibility' section has misspelling in first sentence. Should be "healthy". In addition, there seems to be some font changes in portions of the text.

We have now corrected misspelling ("healthy"), and the font changes.
